# Chinese medicine *Phragmites communis* (Lu Gen) for acute respiratory tract infections: a systematic review and meta-analysis of randomized controlled trials

**DOI:** 10.3389/fphar.2024.1242525

**Published:** 2024-03-06

**Authors:** Min Fang, Ling-Yao Kong, Guang-He Ji, Feng-Lan Pu, You-Zhu Su, Yu-Fei Li, Michael Moore, Merlin Willcox, Jeanne Trill, Xiao-Yang Hu, Jian-Ping Liu

**Affiliations:** ^1^ Centre for Evidence-Based Chinese Medicine, Beijing University of Chinese Medicine, Beijing, China; ^2^ School of Traditional Chinese Medicine, Beijing University of Chinese Medicine, Beijing, China; ^3^ Primary Care and Population Sciences, Faculty of Medicine, University of Southampton, Southampton, United Kingdom; ^4^ Research Design Service South Central, National Institute of Health Research, Southampton, United Kingdom

**Keywords:** *Phragmites australis (Cav.) Trin. ex Steud*, *Phragmites communis*, Chinese herbal medicine, acute respiratory tract infections, systematic review, meta-analysis, randomized controlled trials

## Abstract

**Background:** Acute respiratory tract infections (ARTIs) are the most common cause of morbidity and mortality worldwide, with most people experiencing at least one episode per year. Current treatment options are mainly symptomatic therapy. Antivirals, antibiotics, and glucocorticoids are of limited benefit for most infections. Traditional Chinese medicine has shown potential benefits in the treatment of ARTIs.

**Objective:** The objective of this study was to determine the efficacy, effectiveness, and safety of *Phragmites communis Trin.* (*P. communis, a synonym of Phragmites australis (Cav.) Trin. ex Steud*) as monotherapy or as part of an herb mixture for ARTIs.

**Method:** Eight databases and two clinical trial registries were searched from inception to 8 February 2023 for randomized controlled trials (RCTs) evaluating any preparation involving *P. communis* without language restrictions. The Risk of Bias Tool 2.0 was used to assess the risk of bias of the included trials. RevMan 5.3 software was used for data analyses with effects estimated as risk ratios (RRs), mean differences (MDs), or standardized mean differences (SMDs) with 95% confidence intervals (CIs). The online GRADEpro tool was used to assess the certainty of the evidence, if available.

**Results:** Forty-two RCTs involving 6,879 patients with ARTIs were included, with all trials investigating *P. communis* as part of an herbal mixture. Of the included trials, the majority (38/42) were considered high risk. Compared to the placebo, *P. communis* preparations improved the cure rate [RR = 1.60, 95% CI (1.13, 2.26)] and fever clearance time [MD = −2.73 h, 95% CI (−4.85, −0.61)]. Compared to usual care alone, *P. communis* preparations also significantly improved the cure rate [RR = 1.57, 95% CI (1.36, 1.81)] and fever clearance time [SMD = −1.24, 95% CI (−2.37, −0.11)]. *P. communis* preparations plus usual care compared to usual care alone increased the cure rate [RR = 1.55, 95% CI (1.35, 1.78)], shortened the fever clearance time [MD = −19.31 h, 95% CI (−33.35, −5.27)], and improved FEV1 [ MD = 0.19 L, 95% CI (0.13, 0.26)] and FVC [ MD = 0.16 L, 95% CI (0.03, 0.28)].

**Conclusion:** Low- or very low-certainty evidence suggests that *P. communis* preparations may improve the cure rate of ARTIs, shorten the fever clearance time in febrile patients, and improve the pulmonary function of patients with acute exacerbation of chronic obstructive pulmonary disease or chronic bronchitis. However, these findings are inconclusive and need to be confirmed in rigorously designed trials.

**Systematic review registration**: PROSPERO, identifier CRD42021239936

## Introduction

Acute respiratory tract infections (ARTIs) encompass a variety of respiratory illnesses that can be broadly categorized into acute upper respiratory tract infections and acute lower respiratory tract infections. These infections are primarily caused by viruses or mixed viral–bacterial infections (WHO, 2014). ARTIs manifest in various ways depending on the location of the infection, resulting in symptoms such as fever, runny nose, nasal congestion, cough, dry throat, and muscle aches, which may disrupt the normal functioning of the respiratory system (Chinse Medical Association CMA et al., 2019).

ARTIs represent the leading cause of morbidity and mortality resulting from infectious diseases worldwide, with a particularly pronounced impact on the elderly and children in low-income and middle-income countries. Moreover, ARTIs rank as the most prevalent disease encountered in primary care settings (Renati and Linder, 2016). In the United States, the annual economic burden of influenza alone is estimated at $11.2 billion. In 2017, the global cost of hospitalization and outpatient visits for the treatment of acute lower respiratory tract infections caused by respiratory syncytial virus in children under the age of 5 years was estimated at €4.82 billion, 65% of which occurred in developing countries (Zhang et al., 2020). In 2019, the number of cases of upper respiratory tract infections reached 17.2 billion globally, accounting for 42.82% of all cases of disease and injury in the Global Burden of Disease 2019 study (Jin et al., 2021).

Upper respiratory tract infections are believed to be caused by viral infections in approximately 70%–80% of cases, with viruses responsible for 6%–61% of pathogenic microorganisms in lower respiratory tract infections (Expert Consensus Group On Emergency Diagnosis And Treatment Of Acute Respiratory Viral Infections In Adults, 2021). In the absence of specific antiviral therapeutic drugs, the treatment of ARTIs often focuses on alleviating symptoms (Expert Consensus Group On Emergency Diagnosis And Treatment Of Acute Respiratory Viral Infections In Adults, 2021). However, antibiotics are frequently prescribed and over-used for ARTIs (Hu et al., 2023) despite a lack of evidence supporting their efficacy (Harris et al., 2016). High levels of antibiotic use lead to antibiotic resistance (Goossens et al., 2005), which could lead to 10 million deaths per year by 2050 if no action is taken (O'Neill, 2016). This has prompted medical decision makers, healthcare professionals, and researchers to explore alternative treatments for common infections which do not require antibiotics.

Chinese medicine compound prescriptions refer to a group of Chinese herbs that are thoughtfully formulated based on the principles of composition. The selection of appropriate herbs in accordance with the national standard of concoction and the requirements of the Chinese Pharmacopoeia in precise dosages is guided by Chinese medicine diagnosis and treatment guidance, the holistic concept, and theories of Chinese medicine. A traditional Chinese medicine (TCM) formula consists of different herbs that work together to achieve a therapeutic effect. Each herb in the formula has a specific role to play. The “assistant” herb is one of the four types of herbs in a formula, the others being the “monarch” herb, the “minister” herb, and the “guide” herb (Yao et al., 2013). The assistant herb is used to enhance the therapeutic effects of the monarch and minister herbs and modulate their adverse effects. It can also treat less-important symptoms by its own action (WHO, 2023). These prescriptions have complex chemical compositions and diverse pharmacological effects. TCM is also frequently used to treat ARTIs in China and could be an alternative to antibiotics to reduce antibiotic resistance (Xia et al., 2023).

*Phragmitis rhizoma*, or Lu Gen in Chinese, is the fresh or dried rhizome of the perennial grass *Phragmites communis* Trin, a synonym of *Phragmites australis (Cav.) Trin. ex Steud*. It is widely distributed throughout the world, especially in wetlands, marshes, and lakes. According to the Pharmacopoeia of the People’s Republic of China 2020 (National Pharmacopoeia Committee NPC, 2020), *P. communis* is characterized by its ability to clear heat and reduce fire, nourish yin, and generate fluid. Therefore, *P. communis* is often used to treat heat-related symptoms. As a common traditional Chinese medicine, *P. communis* has been utilized in clinical practice in China for over 2,000 years. The ancient book “*The Famous Doctor’s Book (《名医别录》),*” compiled by Tao Hongjing around the 4th century AD, describes the use of *P. communis* to alleviate thirst related to consumption, reduce fever caused by yin deficiency, and alleviate frequent urination (Tao and Shang, 1986). In the *Treatise on Medicinal Properties (《药性论》)*, it is stated that *P. communis* can relieve high fever and improve appetite (Zhen, 1983). The *Materia Medica Tujing(《本草图经》)* records that *P. communis* can be used to treat lung-heat cough, thick phlegm, pulmonary carbuncle, and coughing up pus and blood (Su and Shang, 1994). In clinical practice, it is often combined with other herbs boiled in water to treat conditions such as colds, halitosis, hepatitis, bronchitis, lung abscesses, and acute tonsillitis (Sun, 2016). In ancient texts, it is regarded as an “assistant” herb used in combination with other herbs.

At present, a variety of Chinese patent medicines containing *P. communis* and with marketing authorization are being applied in clinical practice in China. These include Yin Qiao San, Sangju Ganmao granules (tablets), and antiviral oral liquid, which are mainly used for the treatment of respiratory infections such as colds and flu. Among them, Yin Qiao San, Sangju Ganmao tablets, antiviral oral liquid, Shufeng Jiedu capsules, and Ganmao Qingre granules are widely used in clinical practice and are included in the Pharmacopoeia of the People’s Republic of China 2020 (National Pharmacopoeia Committee NPC, 2020).

To date, 83 compounds have been isolated and identified from *P. communis*, including p-coumaric acid, polysaccharides, vitamin C, vitamin B1, vitamin B2, fatty acids, amino acids, sterols, and polyphenols (Ren et al., 2022). More information is provided in Supplementary Figure S1. *P. communis* exhibits antipyretic, anti-inflammatory, antibacterial, analgesic, and immunomodulatory effects (Sun, 2016; Zuo et al., 2019).

### Antipyretic effects

Oral administration of *P. communis* has a significant antipyretic effect in mice with fever induced by dried yeast. The mechanism of this antipyretic effect is associated with the inhibition of IL-1β, TNF-α, and cAMP expression *in vivo*, as well as the suppression of cyclooxygenase in the hypothalamic temperature center, ultimately reducing the release of PGE2 (Liu et al., 2021).

### Anti-inflammatory effect

Stigmasta-3,5-dien-7-one, derived from *P. communis,* exhibits potent anti-inflammatory activity by suppressing the lipopolysaccharide-stimulated production of NO, PGE2, and cytokines (TNF-α, IL-1β, and IL-6), as well as inhibiting the induction of iNOS and COX-2 protein in lipopolysaccharide-induced RAW 264.7 cells (Park et al., 2016).

### Antibacterial effect

The hydrolysis of *P. communis*-derived oligosaccharides (ROs) using H_2_O_2_ has shown significant antibacterial activity, with a 13.57 mm inhibition zone against *S. aureus* at a concentration of 100 μg/mL (Qian and Jiang, 2014).

### Analgesic effect

Additionally, active ingredients extracted from the above-ground parts of *P. communis using* methanol, petroleum ether, and carbon tetrachloride have demonstrated strong peripheral analgesic activity in Swiss albino mice (Sultan et al., 2017).

### Immune-enhancing effect

Furthermore, *P. communis* has been found to enhance immunity. Gavage administration of fresh *P. communis* aqueous extract increased lymphocyte transformation, NK cell activity, and T-cell immune response function in mice, with more pronounced effects at higher doses (22.5 g/kg) (Sun et al., 2016; Zhang et al., 2016). However, this is a very high dose and is unlikely to be achievable in humans.

Although these findings seem promising, they are all from laboratory experiments (*in vitro* and in animals). It is very important to understand the effectiveness of *P. communis* preparations in human patients because this is key to deciding whether they can be recommended in clinical treatment guidelines. There has been a notable absence of systematic reviews examining the clinical evidence about the efficacy, effectiveness, and safety of *P. communis* in the treatment of ARTIs. This review aims to fill this gap to inform the development of evidence-based clinical guidelines for the treatment of common infections.

## Objectives

The aim of this study was to systematically evaluate the clinical efficacy, effectiveness, and safety of Chinese herbal medicine *P. communis* (Lu Gen) or herbal formulations containing *P. communis* in the treatment of ARTIs in randomized controlled trials (RCTs).

## Methods

### Search methods

We searched eight electronic databases up to 8 February 2023: China National Knowledge Infrastructure (CNKI), Chinese Scientific Journal Database (VIP), Chinese BioMedical Literature Database (CBM), Wanfang Database (Wanfang), PubMed, the Cochrane Library, Embase, and Web of Science. Additionally, we conducted searches on ClinicalTrials.gov and the Chinese Clinical Trial Registry from their inception to 8 February 2023.

Our search terms were adapted to match the requirements of each database. These search terms encompassed “*Phragmites communis*,” “Lu Gen,” and “acute respiratory tract infection,” among others. Further details on additional search terms and strategies in both Chinese and English, tailored to each specific database, can be found in Supplementary Table S1. To identify additional relevant studies, we also reviewed the references of eligible articles. We imposed no language restrictions, and translations were procured whenever necessary.

### Criteria for considering studies for this review

#### Types of studies

RCTs were included. Cross-over trials would be included if comparative data before crossing-over are available.

#### Types of participants

We included patients aged 18 years or older who were diagnosed with ARTIs or presented with characteristic ARTI symptoms, with a symptom duration of less than 4 weeks. A clinical diagnosis of ARTI was the primary inclusion criterion. Our predefined criteria encompassed the following conditions: acute nasopharyngitis, acute sinusitis, acute pharyngitis, acute tonsillitis, acute laryngitis and tracheitis, acute conjunctivitis, acute epiglottitis, acute laryngopharyngitis, acute herpetic pharyngitis, acute bronchitis, common cold, influenza, and acute exacerbation of chronic obstructive pulmonary disease (AECOPD). Cases of novel coronavirus infection and pneumonia were excluded. The most prevalent symptoms associated with ARTIs included fever, nasal congestion, nasal discharge, cough, sore throat, sneezing, itchy throat, fatigue, headache, and muscular soreness.

#### Types of interventions

We included any oral preparation derived from the root of *P. communis*, whether used as monotherapy or as part of an herbal mixture. No restrictions were imposed regarding dosage or treatment duration. Studies that evaluated non-pharmacological therapy, such as massage or acupuncture, were excluded. Additionally, studies were excluded if we could not ascertain the herbal formula or if detailed information could not be obtained from other sources, such as a pharmacopoeia.

Although Shufeng Jiedu capsules met our inclusion criteria, we opted not to include these studies to avoid redundancy, as the data have already been analyzed in previous studies (Zhang et al., 2020; Xia et al., 2020).

#### Types of control

We included studies that compared the treatment in question against no treatment, placebo, or usual treatment, such as antipyretics, antivirals, or antibiotics.

### Pre-specified outcomes included

#### Primary outcomes

The time to the disappearance of the main symptoms or the proportion of patients with the main symptom resolved.

The main symptoms of interest were fever, nasal congestion, nasal discharge, cough, sore throat, sneezing, itchy throat, fatigue, headache, and muscular soreness. The cure rate is the proportion of patients with total or almost total elimination of symptoms of ARTIs, generally 3–5 days after starting treatment.

#### Secondary outcomes

1. TCM syndrome scores

The TCM syndrome score is an index for scoring TCM syndrome and objectively evaluating the efficacy and effectiveness of TCM. It is based on expert experience and begins with a list of primary and secondary symptoms for a specific disease. The weights were determined according to the contribution of the primary and secondary symptoms. Symptoms can generally be classified into four levels, i.e., normal, mildly abnormal, moderately abnormal, and severely abnormal. Finally, the total symptom scores were calculated, and the graded diagnostic criteria of TCM syndrome were classified according to the total scores.

The TCM syndrome score scale for a specific disease is often developed concerning the Chinese medicine clinical research guideline (CMCRG) edited by Zheng (2002).

2. Time spent absent from school or work due to illness
3. Adverse events

We defined serious adverse events according to the guidelines provided by the International Council on Harmonization of Technical Requirements for Registration of Pharmaceuticals for Human Use (ICH). Serious adverse events encompassed any event resulting in death, posing a life-threatening situation, necessitating hospitalization, or leading to persistent or significant disability. This also included abnormalities in biochemistry results, such as electrolytes and liver and kidney function tests. We adapted an emergent approach for assessing additional outcomes, following the principles outlined in the ICH guidelines (ICH, 1995).

Studies that do not report either our main or additional outcomes were excluded.

### Study selection and data extraction

Four authors (MF, LYK, FLP, and GHJ) independently screened the titles and abstracts of all potential studies identified through our searches. When there was uncertainty, insufficient information, or cases of disagreement, we obtained the full texts of articles and then determined eligibility from the full texts. Reasons for excluding articles at the full-text screening stage were recorded. After identifying eligible studies, two authors independently carried out data extraction using a planned data extraction form. In case of a disagreement, it was resolved through negotiation involving another author (JPL). For the included trials, we extracted the following data, as recommended in the Cochrane Handbook for Systematic Reviews of Interventions (Lefebvre et al., 2022):

1) General information: title, first author, publication language, publication year, country, and settings; 2) participants: diagnosis, symptom duration, total number enrolled, and the number in each comparison group, along with baseline characteristics; 3) interventions: herbal CONSORT items, including the herbal medicinal product name, characteristics of the herbal product, quality control, dosage regimen, and quantitative description; 4) follow-up: length of follow-up, reason for dropouts and withdrawals, and the number of participants affected; 5) outcomes reported: mean and standard deviation (SD) for continuous outcomes and the number of events for dichotomous outcomes; and 6) adverse events.

### Assessment of risk of bias in included studies

MF, LYK, YFL, and YZS were responsible for the risk of bias assessment. For each trial, two authors independently assessed the risk of bias using the Cochrane Collaboration Risk of Bias tool 2.0 (Higgins et al., 2022). Disagreements were discussed and resolved regarding the original protocol and, if necessary, arbitration by another author (XYH).

The Risk of Bias Tool 2.0 is a structured tool for assessing the risk of bias in RCTs. It is designed to guide systematic reviewers in conducting meaningful, outcome-based assessments of trial design, implementation, and reporting. The tool is divided into five fixed bias domains: bias arising from the randomization process, bias due to deviations from intended interventions, bias due to missing outcome data, bias in the measurement of the outcome, and bias in the selection of the reported result. By using this tool, reviewers can identify potential sources of bias and evaluate the overall reliability of the study.

### Measures of treatment effect

We used RevMan 5.3 software for data analysis. Risk ratios (RRs) with corresponding 95% confidence intervals (CIs) were computed for dichotomous variables, while mean differences (MDs) with 95% CI were calculated for continuous data. In cases where outcome measures had consistent units across studies, we reported the effects as standardized mean differences (SMDs).

RevMan 5.3 software is a piece of software dedicated for creating and managing Cochrane systematic reviews. It provides a user-friendly interface for conducting meta-analyses, generating forest plots, and presenting the results in a clear and concise manner.

### Unit of analysis issues

In the trials, we separated the arms into different comparisons to avoid double-counting participants with multiple intervention groups that met the inclusion criteria.

### Dealing with missing data

We proactively reached out to investigators or authors to validate critical study details and obtain missing numerical outcome data when needed (*e.g.*, when a study reported outcomes with a line chart). Where standard deviation was not reported by means, it was calculated from the information reported, such as CIs or p-values. When we did not get a response, we only used the available data in the analyses.

### Assessment of heterogeneity

We assessed between-study heterogeneity using the *I* ^*2*^ statistic, which quantifies the percentage of variation across studies attributed to heterogeneity rather than chance. The rule of thumb for the interpretation of this statistic indicated that *I* ^*2*^ > 30% signifies moderate heterogeneity, *I* ^*2*^ > 50% indicates substantial heterogeneity, and *I* ^*2*^ > 75% suggests considerable heterogeneity. When *I* ^*2*^ values were above 50% for primary outcomes, we conducted a sensitivity analysis to explore potential sources of heterogeneity and factored these findings into our interpretation of the results.

### Assessment of reporting biases

We conducted Egger tests using R software version 4.2.2 to explore potential reporting bias, where applicable, and when a sufficient number of studies were available within a single meta-analysis.

### Data synthesis

Where possible, we planned to conduct our analyses based on intention-to-treat (ITT) data for each outcome, which include data from all randomized participants in the individual trials, regardless of any deviations from the original study plan. When possible, we extracted the end-of-treatment scores for continuous outcomes rather than relying on the change-from-baseline score. Given the anticipated variability in the populations and interventions across the included trials, we used a generic inverse variance random-effects model to pool the data, allowing for the incorporation of heterogeneity into our analysis.

### Subgroup analysis and investigation of heterogeneity

Subgroup analyses were performed for the primary outcomes where there were sufficient studies in each comparison group:

1. *P. communis* as a monotherapy *versus* being part of an herbal mixture
2. ARTI types regarding pathogen (bacterial infection or virus infection)
3. Comparisons with various types of control medications (symptomatic drugs and antimicrobial drugs)

### Sensitivity analysis

We performed sensitivity analyses for the primary outcome to determine whether the conclusions would change when limiting eligibility to trials with a low risk of overall bias. In cases of significant heterogeneity, we planned to perform sensitivity analysis to explore potential sources of heterogeneity in more detail.

#### Certainty assessment of the evidence

The online GRADEpro tool was used to assess the certainty of the evidence, if available.

#### Deviation from the protocol

*P. communis* is a component of numerous Chinese medicine compound prescriptions, but it is seldom studied in isolation. Therefore, conducting an analysis solely on *P. communis* was not feasible. Additionally, due to the substantial number of RCTs involving *P. communis*, we specifically concentrated on RCTs that examined the effectiveness of preparations containing it on ARTIs in adult populations.

## Results

### Results of the search

**FIGURE 1:**
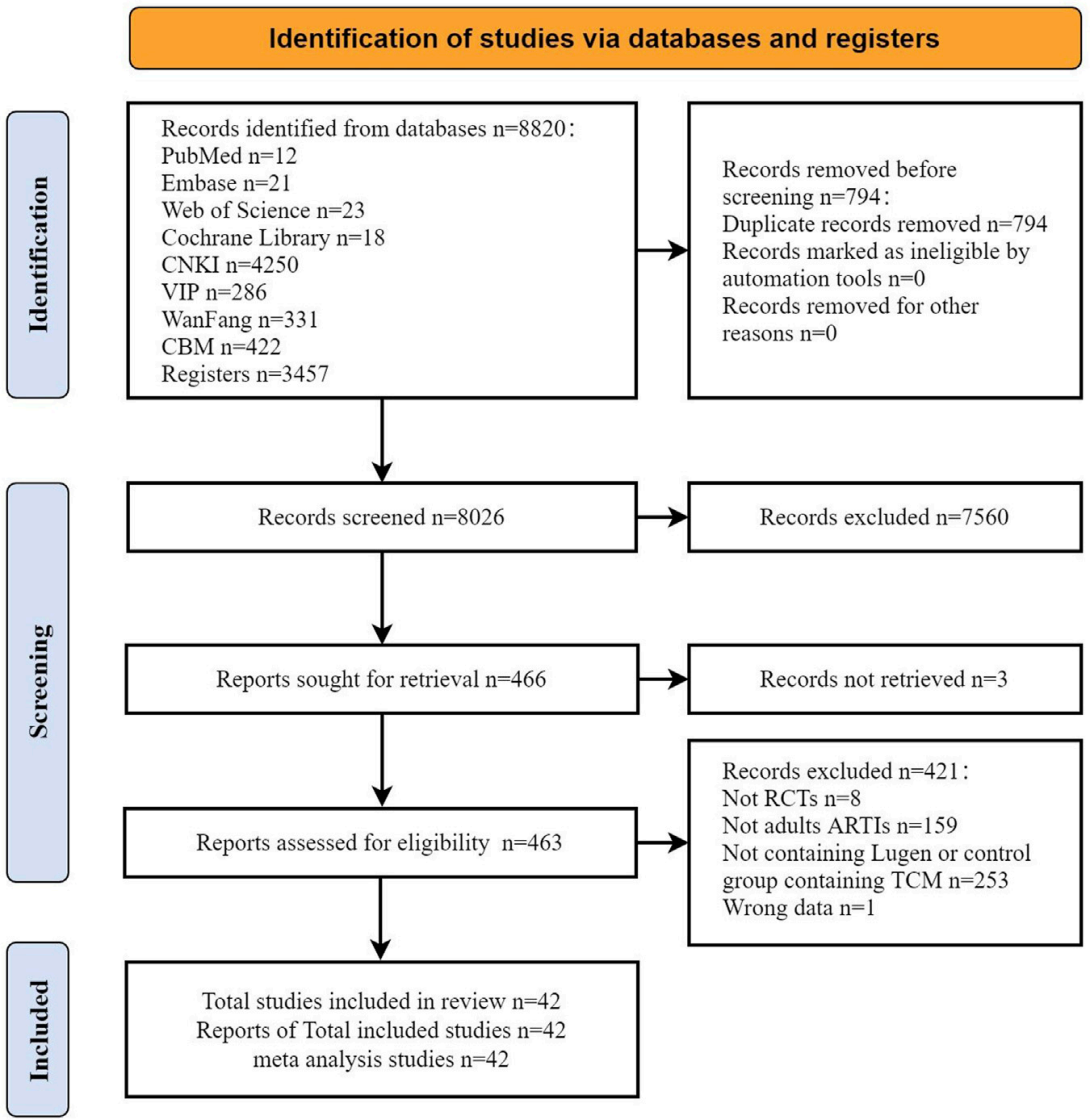
PRISMA flowchart and trial inclusion.

A total of 8,820 records were obtained. After removing 794 duplicates, 8,026 records remained and were screened by title and abstract. Then, 463 records were assessed in full text. A total of 421 studies were excluded for not meeting the inclusion criteria as they were not RCTs (*n* = 8), did not involve adults with ARTIs (*n* = 159), did not contain Lu Gen in the intervention group or contained TCM in the control group (*n* = 253), or had wrong data (*n* = 1). Finally, 42 trials involving 6,879 participants, published between 2006 and 2022, met the inclusion criteria and were included for meta-analysis (Zhang, 2006; Wang, 2007; Guo, 2009; Huai, 2009; Shi et al., 2010; Wang, 2010; Hui et al., 2012; Wong et al., 2012; Xue, 2012; Xu and Zhi, 2013; Zhou, 2013; Sun, 2014a; Sun, 2014b; Chen, 2014; Pan, 2014; Wei, 2014; Xu, 2014; Chang, 2016; Chen and Li, 2016; Cheng et al., 2016; Zhang, 2016; Zhang et al., 2016; Guo and Xie, 2017; Xue et al., 2017; Zhang, 2017; Bi et al., 2019; Hu, 2019; Luo and Zhong, 2019; Yang, 2019; Zheng, 2019; Hong et al., 2020; Li, 2020; Wang, 2020; Ye et al., 2020; Song, 2021; Zhou and Qu, 2021; Li et al., 2022a; Li et al., 2022b; Li et al., 2022c; Li et al., 2022d; Sun, 2022). Two trials were published in English, and the remaining were in Chinese (Figure 1). Figure 1 shows the PRISMA flowchart and trial inclusion.

### Description of the included trials

The included studies were conducted in patients with AECOPD (*n* = 13) (Song, 2021; Li, 2020; Zhang, 2017; Wei, 2014; Li et al., 2022a; Li et al., 2022b; Zheng, 2019; Zhang et al., 2016; Chen, 2014; Shi et al., 2010; Hong et al., 2020; Wang, 2020; Chang, 2016), acute bronchitis (*n* = 2) (Bi et al., 2019; Ye et al., 2020), influenza (*n* = 6) (Wang, 2007; Hui et al., 2012; Sun, 2014b; Zong and Chen, 2020; Li et al., 2022c; Li et al., 2022d), acute upper respiratory infections (*n* = 21) (Zhang, 2006; Guo, 2009; Huai, 2009; Wang, 2010; Wong et al., 2012; Xue, 2012; Xu and Zhi, 2013; Zhou, 2013; Sun, 2014a; Pan, 2014; Xu, 2014; Chen and Li, 2016; Cheng et al., 2016; Zhang, 2016; Guo and Xie, 2017; Xue et al., 2017; Hu, 2019; Luo and Zhong, 2019; Yang, 2019; Zhou and Qu, 2021; Li et al., 2022c), and acute respiratory infection with fever (Sun, 2022). A total of 16 studies (Zhang, 2006; Huai, 2009; Wong et al., 2012; Xu and Zhi, 2013; Zhou, 2013; Sun, 2014a; Sun, 2014b; Pan, 2014; Cheng et al., 2016; Xue et al., 2017; Yang, 2019; Zong and Chen, 2020; Li et al., 2022a; Li et al., 2022c; Li et al., 2022d; Sun, 2022) included patients with onset within 48 h. A total of 13 studies (Wang, 2007; Guo, 2009; Shi et al., 2010; Wang, 2010; Xue, 2012; Chen, 2014; Wei, 2014; Xu, 2014; Chen and Li, 2016; Bi et al., 2019; Hu, 2019; Luo and Zhong, 2019; Zhou and Qu, 2021) included patients with the onset of illness within 3–23 days, and 13 trials (Hui et al., 2012; Chang, 2016; Zhang, 2016; Zhang et al., 2016; Guo and Xie, 2017; Zhang, 2017; Zheng, 2019; Hong et al., 2020; Li, 2020; Wang, 2020; Ye et al., 2020; Song, 2021; Li et al., 2022b) did not report the details of onset.

The mixtures containing *P. communis* included Chinese patent medicines (*n* = 7) (Huai, 2009; Wong et al., 2012; Xue, 2012; Zhou, 2013; Pan, 2014; Sun, 2014a; Li et al., 2022c), fixed Chinese herbal medicine formulas (*n* = 25) (Wang, 2007; Shi et al., 2010; Xu and Zhi, 2013; Sun, 2014a; Sun, 2014b; Chen, 2014; Wei, 2014; Xu, 2014; Chang, 2016; Cheng et al., 2016; Zhang, 2016; Zhang et al., 2016; Xue et al., 2017; Zhang, 2017; Bi et al., 2019; Hu, 2019; Yang, 2019; Zheng, 2019; Li, 2020; Wang, 2020; Zong and Chen, 2020; Song, 2021; Li et al., 2022d; Li et al., 2022b; Sun, 2022), and individualized Chinese herbal medicine formulas (n = 11) (Zhang, 2006; Guo, 2009; Wang, 2010; Hui et al., 2012; Chen and Li, 2016; Guo and Xie, 2017; Luo and Zhong, 2019; Hong et al., 2020; Ye et al., 2020; Zhou and Qu, 2021; Li et al., 2022a).

Six trials (Wong et al., 2012; Zhou, 2013; Cheng et al., 2016; Xue et al., 2017; Yang, 2019; Li et al., 2022c) compared *P. communis versus* the placebo. Twenty trials (Shi et al., 2010; Wang, 2010; Sun, 2014b; Chen, 2014; Wei, 2014; Xu, 2014; Zong and Chen, 2020; Chang, 2016; Zhang et al., 2016; Zhang, 2017; Bi et al., 2019; Zheng, 2019; Hong et al., 2020; Li, 2020; Wang, 2020; Ye et al., 2020; Song, 2021; Li et al., 2022a; Li et al., 2022b; Sun, 2022) compared *P. communis* plus usual care *versus* usual care. Sixteen trials (Zhang, 2006; Wang, 2007; Guo, 2009; Huai, 2009; Hui et al., 2012; Xue, 2012; Xu and Zhi, 2013; Sun, 2014a; Pan, 2014; Chen and Li, 2016; Zhang, 2016; Guo and Xie, 2017; Hu, 2019; Luo and Zhong, 2019; Zhou and Qu, 2021; Li et al., 2022d) compared *P. communis versus* usual care. Seven (Guo, 2009; Hui et al., 2012; Xue, 2012; Sun, 2014a; Xu, 2014; Li et al., 2022c; Li et al., 2022b) of the included studies allowed adjuvant treatments, such as oral acetaminophen, if the patient had a temperature ≥39°C.

Three included studies (Hui et al., 2012; Xue, 2012; Song, 2021) reported the time to disappearance of the main symptoms (cure time). A total of 36 studies (Zhang, 2006; Wang, 2007; Guo, 2009; Shi et al., 2010; Wang, 2010; Hui et al., 2012; Xu and Zhi, 2013; Zhou, 2013; Sun, 2014a; Sun, 2014b; Chen, 2014; Pan, 2014; Wei, 2014; Xu, 2014; Chang, 2016; Chen and Li, 2016; Cheng et al., 2016; Zhang, 2016; Zhang et al., 2016; Guo and Xie, 2017; Xue et al., 2017; Zhang, 2017; Bi et al., 2019; Hu, 2019; Luo and Zhong, 2019; Yang, 2019; Zheng, 2019; Hong et al., 2020; Li, 2020; Ye et al., 2020; Zong and Chen, 2020; Song, 2021; Zhou and Qu, 2021; Li et al., 2022a; Li et al., 2022d; Sun, 2022) reported the proportion of patients with symptoms resolved (cure rate). Seven studies (Sun, 2014a; Zhang, 2006; Xu, 2014; Cheng et al., 2016; Zhang, 2016; Yang, 2019; Zong and Chen, 2020; Sun, 2022) reported on the cooling onset time. Ten studies (Zhang, 2006; Hui et al., 2012; Xue, 2012; Sun, 2014a; Xu, 2014; Cheng et al., 2016; Zhang, 2016; Yang, 2019; Zong and Chen, 2020; Sun, 2022) reported on the fever clearance time. However, only few studies have reported definitions of the cooling onset time and the fever clearance time. Three studies (Zhang, 2006; Cheng et al., 2016; Sun, 2022) defined the cooling onset time as the time it takes for the body temperature to decrease by 0.5°C after the initiation of medication. Two studies (Hui et al., 2012; Sun, 2014b) defined the fever clearance time as the time it takes for the medication to be administered until the body temperature drops to normal and can be maintained for more than 24 h, whereas three studies (Zhang, 2006; Cheng et al., 2016; Sun, 2022) defined the fever clearance time as the time from the administration of the drug until the body temperature drops to normal. The rest of the studies did not give detailed information.

No trials reported time spent absent from school or work due to illness. Twelve studies (Zhou, 2013; Sun, 2014a; Sun, 2014b; Wei, 2014; Zhang et al., 2016; Guo and Xie, 2017; Zhang, 2017; Li, 2020; Li et al., 2022a; Li et al., 2022b; Li et al., 2022d; Sun, 2022) reported TCM syndrome scores about the CMCRG. The primary and secondary symptoms of the scales differed slightly in each study due to the specific disease. The reported scales all included respiratory symptoms, mainly cough, sputum, runny nose, nasal congestion, fever, sore throat, and physical discomfort. There was also some variation in the scores for each symptom across the scales.

Sixteen studies (Zhang, 2006; Huai, 2009; Wong et al., 2012; Zhou, 2013; Sun, 2014b; Pan, 2014; Wei, 2014; Cheng et al., 2016; Zhang et al., 2016; Zhang, 2017; Wang, 2020; Ye et al., 2020; Zhou and Qu, 2021; Li et al., 2022a; Li et al., 2022d; Li et al., 2022c) reported adverse events. Some studies also focused on lung function and inflammation-related indicators. All the outcomes were measured during or after treatment. The duration of treatment was 2 days to 4 weeks.

Seven (Zhou, 2013; Sun, 2014a; Sun, 2014b; Wei, 2014; Zhang, 2017; Li, 2020; Sun, 2022) of 42 reports were degree theses. More information about the characteristics of the included studies is provided in Supplementary Table S2.

Seven studies (Sun, 2014a; Wong et al., 2012; Zhou, 2013; Cheng et al., 2016; Xue et al., 2017; Yang, 2019; Li et al., 2022a) used Chinese patent medicines, including four listed medicines, namely, Ganmao Qingre granules, antiviral oral liquid, Siji antiviral mixture, and Yin Qiao San, and two in-hospital preparations: Qingre Huashi oral liquid and Qingjie Kanggan granules. Ganmao Qingre granules, an antiviral oral liquid, and Yin Qiao San are included in the Pharmacopoeia of the People’s Republic of China 2020. The pharmacopoeia provides a detailed record of all the ingredients and the exact manufacturing process for these medicines. Detailed information is provided in Supplementary Table S3.

Twenty-six trials used fixed Chinese herbal medicine formulas and reported their composition. Only five of these trials reported the origin of the TCM compound prescriptions, three of which were from classical compound prescriptions, i.e., The Essentials of the Golden Chamber, The Essentials of Thousand Gold, and The Woman’s Good Prescription, and the other two were from the empirical prescriptions of Prof. J.J. Chen and Prof. Q.N. Wan, respectively. No studies reported on the authentication of each ingredient. Twenty-five studies reported on the rationale for the composition of the herbal compound. Only eight studies reported pharmacological or toxicological related tests. Detailed information is provided in Supplementary Table S4.

Eleven trials used individual Chinese herbal medicine formulas and reported the composition and principle of the formulation. Only one trial showed *Phragmitis rhizoma* as an assistant medicinal. One study did not report the method of preparation. The rest of the formulations were either decocted in water or supplied directly by the pharmacy. No studies were reporting on the safety assessment of Chinese medicine compounding. Detailed information is provided in Supplementary Table S5.

The authentication methods, chemical analysis, and quality control of each ingredient were not reported for Chinese patent medicines, fixed Chinese herbal medicine formulas, and individual Chinese herbal medicine formulas.

### Risk of bias in included studies

**FIGURE 2:**
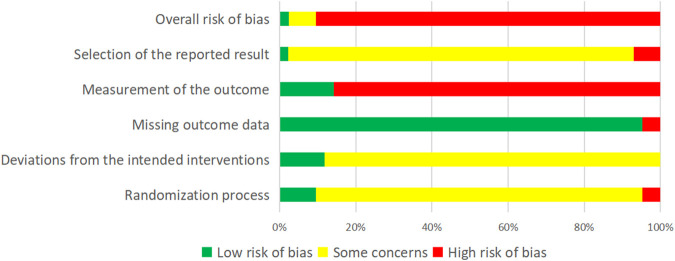
Risk of bias of one of the important outcomes for all included studies.

We selected one outcome from each trial that we considered important to evaluate. One of the 42 studies was at low risk of bias; 3 studies had some concerns; and 38 studies were assessed as high risk. Detailed information is shown in Figure 2.

#### Domain 1: the randomization process

All studies mentioned randomization, 20 studies (Zhang, 2006; Wang, 2007; Guo, 2009; Shi et al., 2010; Wang, 2010; Hui et al., 2012; Xue, 2012; Xu and Zhi, 2013; Chen, 2014; Pan, 2014; Chang, 2016; Chen and Li, 2016; Zhang, 2016; Guo and Xie, 2017; Xue et al., 2017; Hu, 2019; Luo and Zhong, 2019; Zheng, 2019; Zong and Chen, 2020; Li et al., 2022b) did not report further on specific randomization methods, 20 studies (Huai, 2009; Wong et al., 2012; Zhou, 2013; Sun, 2014a; Song, 2021; Wei, 2014; Xu, 2014; Cheng et al., 2016; Zhang et al., 2016; Zhang, 2017; Bi et al., 2019; Yang, 2019; Hong et al., 2020; Li, 2020; Wang, 2020; Ye et al., 2020; Zhou and Qu, 2021; Li et al., 2022a; Li et al., 2022c; Sun, 2022) used random number tables, 1 study (Li et al., 2022d) used lottery, and 1 study (Sun, 2014b) randomized according to odd and even numbers in the order of visits.

One study (Sun, 2014a) used randomized envelopes, and three studies (Wong et al., 2012; Xue et al., 2017; Li et al., 2022c) conducted multicenter randomized controlled double-blind trials and were considered to have performed allocation concealment. The remaining studies did not report allocation concealment.

Only one study (Huai, 2009) did not report a post-randomization baseline, and 41 studies reported a balanced comparable baseline after random assignment.

#### Domain 2: deviations from intended interventions

Thirty-six studies (Zhang, 2006; Wang, 2007; Guo, 2009; Zong and Chen, 2020; Huai, 2009; Shi et al., 2010; Wang, 2010; Hui et al., 2012; Xue, 2012; Xu and Zhi, 2013; Zhou, 2013; Xue et al., 2017; Chen, 2014; Pan, 2014; Wong et al., 2012; Xu, 2014; Chang, 2016; Chen and Li, 2016; Zhang, 2016; Zhang et al., 2016; Guo and Xie, 2017; Bi et al., 2019; Hu, 2019; Luo and Zhong, 2019; Yang, 2019; Zheng, 2019; Hong et al., 2020; Li, 2020; Wang, 2020; Ye et al., 2020; Song, 2021; Zhou and Qu, 2021; Li et al., 2022a; Li et al., 2022d; Sun, 2022; Li et al., 2022c) used intention-to-treat analysis, and six studies (Sun, 2014a; Sun, 2014b; Wei, 2014; Cheng et al., 2016; Zhang, 2017; Li et al., 2022b) used per-protocol analysis. Six studies (Wong et al., 2012; Zhou, 2013; Cheng et al., 2016; Xue et al., 2017; Yang, 2019; Li et al., 2022c) were placebo-controlled double-blind trials, so the participants, intervention providers, and caregivers were all unaware of the interventions the subjects received. Thirty-six trials (Zhang, 2006; Wang, 2007; Guo, 2009; Huai, 2009; Shi et al., 2010; Wang, 2010; Hui et al., 2012; Xue, 2012; Xu and Zhi, 2013; Sun, 2014a; Sun, 2014b; Chen, 2014; Pan, 2014; Wei, 2014; Xu, 2014; Chang, 2016; Chen and Li, 2016; Zhang, 2016; Zhang et al., 2016; Guo and Xie, 2017; Zhang, 2017; Bi et al., 2019; Hu, 2019; Luo and Zhong, 2019; Zheng, 2019; Hong et al., 2020; Li, 2020; Wang, 2020; Ye et al., 2020; Zong and Chen, 2020; Song, 2021; Zhou and Qu, 2021; Li et al., 2022a; Li et al., 2022b; Li et al., 2022d; Sun, 2022) were not blinded, so it is likely that participants and researchers were aware of the interventions the subjects received. Thirty-one studies (Zhang, 2006; Wang, 2007; Guo, 2009; Huai, 2009; Shi et al., 2010; Wang, 2010; Hui et al., 2012; Xue, 2012; Xu and Zhi, 2013; Chen, 2014; Pan, 2014; Xu, 2014; Chang, 2016; Chen and Li, 2016; Zhang, 2016; Zhang et al., 2016; Guo and Xie, 2017; Bi et al., 2019; Hu, 2019; Luo and Zhong, 2019; Zheng, 2019; Hong et al., 2020; Li, 2020; Wang, 2020; Ye et al., 2020; Zong and Chen, 2020; Song, 2021; Zhou and Qu, 2021; Li et al., 2022a; Li et al., 2022c; Sun, 2022) had no evidence of deviation from the intended interventions. One study (Sun, 2014a) divided into eight groups based on the dialectical typing of colds, and the results of the study only reported the overall number of losses to follow-up without specifying the number of dropouts in a particular group. Four studies (Sun, 2014a; Wei, 2014; Cheng et al., 2016; Zhang, 2017) reported reasons for loss to follow-up, but it was impossible to determine the impact of this on outcomes.

#### Domain 3: missing outcome data

Only two studies (Wong et al., 2012; Li et al., 2022c) lost less than 10% to follow-up. The remaining studies compared with the methods section of the published article. A total of 34 studies (Zhang, 2006; Wang, 2007; Guo, 2009; Zong and Chen, 2020; Huai, 2009; Shi et al., 2010; Wang, 2010; Hui et al., 2012; Xue, 2012; Xu and Zhi, 2013, Song, 2021; Zhou, 2013; Chen, 2014; Pan, 2014; Xu, 2014; Chang, 2016; Chen and Li, 2016; Zhang, 2016; Zhang et al., 2016; Guo and Xie, 2017; Xue et al., 2017; Bi et al., 2019; Hu, 2019; Luo and Zhong, 2019; Yang, 2019; Zheng, 2019; Hong et al., 2020; Li, 2020; Wang, 2020; Ye et al., 2020; Zhou and Qu, 2021; Li et al., 2022a; Li et al., 2022d; Sun, 2022) did not report the number of patients lost to follow-up. Four studies (Sun, 2014a; Sun, 2014b; Wei, 2014; Li et al., 2022b) reported that less than 10% were lost to follow-up, and two studies lost more than 10% to follow-up (Cheng et al., 2016; Zhang, 2017), with no evidence that the missing data did not cause bias and no information on whether the missing data were related to the true values.

#### Domain 4: measurement of the outcome

Six studies (Wong et al., 2012; Zhou, 2013; Cheng et al., 2016; Xue et al., 2017; Yang, 2019; Li et al., 2022c) were blinded, and the outcome measures may not have caused bias. The assessors of the remaining 36 studies (Zhang, 2006; Guo, 2009; Huai, 2009; Shi et al., 2010; Wang, 2010; Wang, 2007; Hui et al., 2012; Xue, 2012; Xu and Zhi, 2013; Sun, 2014a; Sun, 2014b; Chen, 2014; Pan, 2014; Wei, 2014; Xu, 2014; Chang, 2016; Chen and Li, 2016; Zhang, 2016; Zhang et al., 2016; Guo and Xie, 2017; Zhang, 2017; Bi et al., 2019; Hu, 2019; Luo and Zhong, 2019; Zheng, 2019; Hong et al., 2020; Li, 2020; Wang, 2020; Ye et al., 2020; Zong and Chen, 2020; Song, 2021; Zhou and Qu, 2021; Li et al., 2022a; Li et al., 2022b; Li et al., 2022c; Sun, 2022) may have been aware of the interventions that patients received, and the outcomes evaluated may have been influenced by subjective judgment.

#### Domain 5: selection of the reported result

Study protocols were obtained for two studies (Wong et al., 2012; Li et al., 2022c), one (Wong et al., 2012) of which was consistent with the protocol, and the other (Li et al., 2022c) had selective reporting. The methods section of the original text of 40 studies was compared with the results; 38 studies were consistent with the results, and 2 studies (Huai, 2009; Wang, 2010) were selectively reported.

### Effects of interventions

#### *P. communis versus* the placebo (six trials)

Six studies (Wong et al., 2012; Zhou, 2013; Cheng et al., 2016; Xue et al., 2017; Yang, 2019; Li et al., 2022c) involved 3,433 participants with symptoms lasting less than 48 h, involving formulations of Waigan Qingre Jiedu formula, Qingre Kanggan granules, Yin Qiao San, and an antiviral oral liquid.

#### Symptom outcomes

**FIGURE 3:**
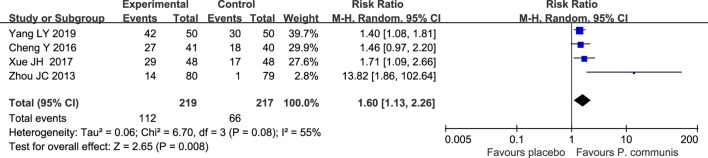
Forest plot of the cure rate in *P. communis* preparations *versus* the placebo.

The results of four studies (Zhou, 2013; Cheng et al., 2016; Xue et al., 2017; Yang, 2019) involving 436 participants combined showed a significant improvement in cure rates in the *P. communis* group compared to the placebo [RR = 1.60, 95% CI (1.13, 2.26)]. See Figure 3.

**FIGURE 4:**
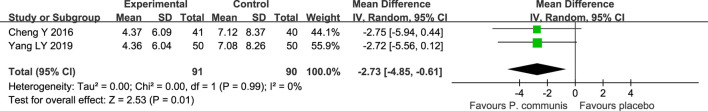
Forest plot of the fever clearance time in *P. communis* preparations *versus* the placebo.

Two studies (Cheng et al., 2016; Yang, 2019) involving 181 participants found a significant reduction in the time to fever reduction [MD = −2.73 h, 95% CI (−4.85, −0.61)] in the *P. communis* group compared to the placebo arm. See Figure 4.

One study (Zhou, 2013) included 159 participants who reported TCM syndrome scores and found that *P. communis* preparations significantly reduced TCM syndrome scores [MD = −6.88, 95% CI (−8.91, −4.85)]. See Supplementary Table S6 for more information.

#### Other outcomes

One study (Wong et al., 2012) conducted in Hong Kong, China, involving 165 participants reported quality of life scores and SF-36 scores, and there were no significant differences in each domain score for the *P. communis* preparation compared to the placebo.

#### *P. communis* plus *usual care versus* usual care alone (20 trials)

A total of 20 studies (Shi et al., 2010; Wang, 2010; Sun, 2014b; Chen, 2014; Wei, 2014; Xu, 2014; Chang, 2016; Zhang et al., 2016; Zhang, 2017; Bi et al., 2019; Zheng, 2019; Hong et al., 2020; Li, 2020; Wang, 2020; Ye et al., 2020; Zong and Chen, 2020; Song, 2021; Li et al., 2022a; Li et al., 2022b; Sun, 2022) involved 1,570 participants, of which 18 studies (Shi et al., 2010; Wang, 2010; Chen, 2014; Wei, 2014; Xu, 2014; Chang, 2016; Zhang et al., 2016; Zhang, 2017; Bi et al., 2019; Zheng, 2019; Hong et al., 2020; Li, 2020; Wang, 2020; Ye et al., 2020; Song, 2021; Li et al., 2022a; Li et al., 2022b; Sun, 2022) had a control group whose treatment included the administration of antimicrobials, symptomatic treatment, and supportive treatment, such as the administration of oxygen and nutrition as recommended by the guidelines, 1 study (Zong and Chen, 2020) gave antivirals, and another study (Sun, 2014b) gave only ibuprofen.

#### Symptom outcomes

**FIGURE 5:**
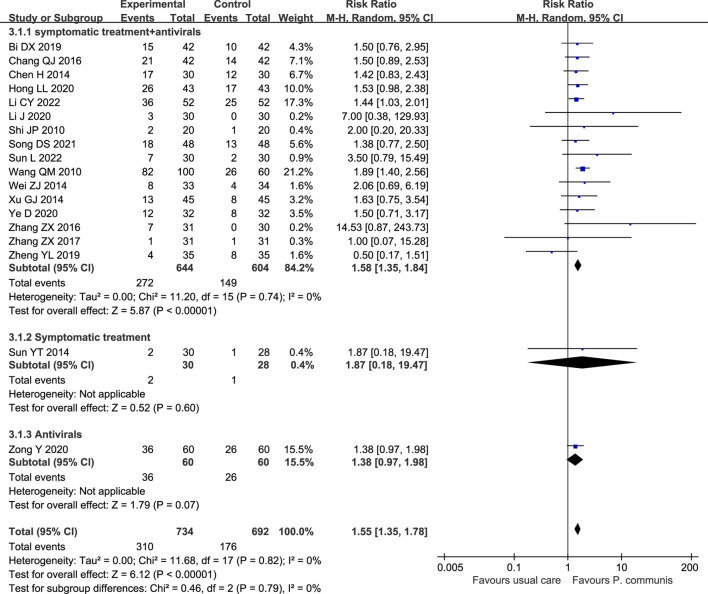
Forest plot of the cure rate in *P. communis* preparations plus usual care *versus* usual care.

A total of 18 trials (Wang, 2010; Shi et al., 2010; Chen, 2014; Wei, 2014; Xu, 2014; Chang, 2016; Zhang et al., 2016; Zhang, 2017; Bi et al., 2019; Zheng, 2019; Hong et al., 2020; Li, 2020; Ye et al., 2020; Song, 2021; Li et al., 2022a; Sun, 2022) involved 1,426 participants, and the meta-analysis showed that *P. communis* plus usual care significantly improved the cure rate [RR = 1.55, 95% CI (1.35, 1.78)]. See Figure 5.

**FIGURE 6:**
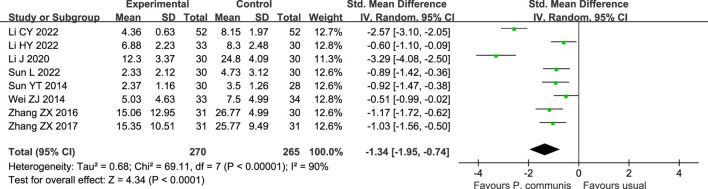
Forest plot of the TCM syndrome score in *P. communis* preparations plus usual care *versus* usual care.

A total of 535 participants in eight studies (Sun, 2014b; Wei, 2014; Zhang et al., 2016; Zhang, 2017; Li, 2020; Li et al., 2022a; Li et al., 2022b; Sun, 2022) reported that *P. communis* plus usual care significantly reduced the TCM syndrome score [n = 8, SMD = −1.34, 95% CI (−1.95, −0.74)]. See Figure 6.

**FIGURE 7:**
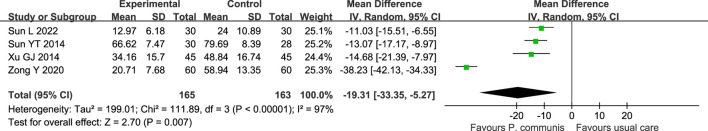
Forest plot of the fever clearance time in *P. communis* preparations plus usual care *versus* usual care.

In addition, *P. communis* preparation plus usual care also significantly reduced the time to fever reduction [n = 4, MD = −19.31 h, 95% CI (−33.35, −5.27)] compared to the control group. Heterogeneity decreased when we removed the study of Zong Y. This study had a larger sample size compared to the other three studies, so we consider the sample size as a source of heterogeneity. See Figure 7.

#### Pulmonary function-related outcomes

*P. communis* preparation plus usual care significantly improved FEV1 [*n* = 6, MD = 0.19 L, 95% CI (0.13, 0.26)], FEV1% [*n* = 7, MD = 4.22%, 95% CI (2.84, 5.60)], FVC [*n* = 6, MD = 0.16 L, 95% CI (0.03, 0.28), and FEV1/FVC [*n* = 7, SMD = 0.82, 95% CI (0.24, 1.40)] scores for AECOPD patients. See Supplementary Table S7 for more information.

#### Inflammatory factor outcomes

*P. communis* preparation plus usual care significantly decreased TNF-α, TGF-β, GRO-α (growth-regulated oncogene alpha, significantly upregulated in various inflammatory diseases), IL-4, IL-6, IL-8, PCT, and WBC and increased IL-10 and LYM%, while for CRP, NEUT%, and SAA (serum amyloid A, a more sensitive marker of inflammation than C-reactive protein), no statistically significant difference was shown between the two groups. See Supplementary Table S7 for more information.

#### Other outcomes

Two studies (Sun, 2014b; Zong and Chen, 2020) involving 178 participants found a significant increase in the rate of influenza virus nucleic acid clearance with *P. communis* preparation in the control group. This was indicated by the detection of influenza virus nucleic acid conversion in patients through reverse transcription–polymerase chain reaction (RT–PCR). Two studies (Li, 2020; Li et al., 2022a) involving 164 participants found a significant decrease in CAT scores (COPD assessment test, a questionnaire designed for people with chronic obstructive pulmonary disease (COPD) to measure the impact of COPD on a person’s life and how this changes over time). One study (Hong et al., 2020) with 86 participants found a significant improvement in quality of life with *P. communis* plus usual care. See Supplementary Table S7 for more information.

#### P. communis versus usual care (16 trials)

##### Symptom outcomes

**FIGURE 8:**
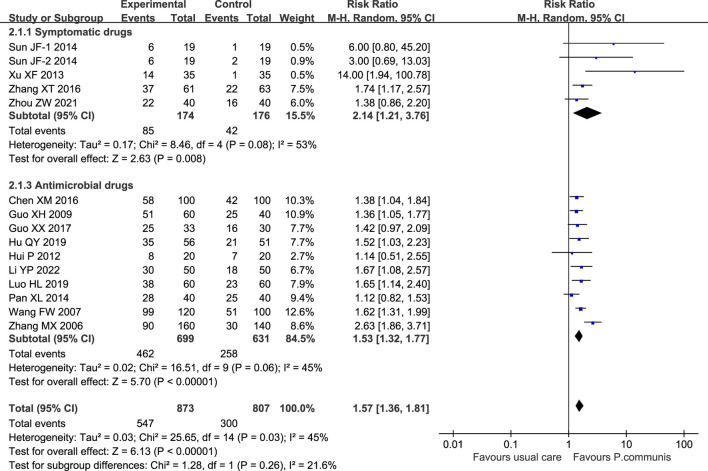
Forest plot of the cure rate in *P. communis* preparations *versus* usual care.

Fourteen studies (Zhang, 2006; Wang, 2007; Guo, 2009; Hui et al., 2012; Xu and Zhi, 2013; Sun, 2014a; Pan, 2014; Chen and Li, 2016; Zhang, 2016; Guo and Xie, 2017; Hu, 2019; Luo and Zhong, 2019; Zhou and Qu, 2021; Li et al., 2022d) involving 1,680 participants reported cure rates, and meta-analysis found that *P. communis* preparation significantly increased the cure rate compared to usual care [RR = 1.57, 95% CI (1.36, 1.81)]. See Figure 8.

**FIGURE 9:**
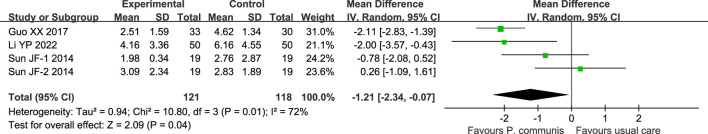
Forest plot of the TCM syndrome score in *P. communis* preparations *versus* usual care.

Three studies (Sun, 2014a; Guo and Xie, 2017; Li et al., 2022a) involving 239 patients with *I* ^*2*^ = 72% and meta-analysis using a random-effects model found that *P. communis* preparations significantly reduced the TCM syndrome score compared to usual care [MD = −1.21, 95% CI (−2.34, −0.07)]. See Figure 9.

**FIGURE 10:**
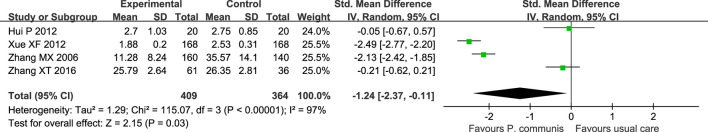
Forest plot of the fever clearance time in *P. communis* preparations versus usual care.

Four studies (Zhang, 2006; Hui et al., 2012; Xue, 2012; Zhang, 2016) involving 773 patients with *I* ^*2*^ = 97% and meta-analysis using a random-effects model found that *P. communis* preparations significantly reduced the fever clearance time compared to Western treatment [SMD = −1.24, 95% CI (−2.37, −0.11)]. Two of these studies were on influenza, and two were on colds. Because of the high heterogeneity, we explored the sources of heterogeneity using subgroup analysis, and when stratified by sample size, the heterogeneity decreased and the results were different, so we suggest that sample size may be one of the sources of heterogeneity. Also, the difference in measurement frequency may be a source of heterogeneity due to the inconsistent units of time to fever reduction, with some using days and others using hours. See Figure 10.

**FIGURE 11:**
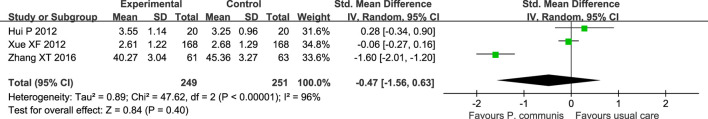
Forest plot of the cure time in *P. communis* preparations *versus* usual care.

A total of 500 participants in three studies (Hui et al., 2012; Xue, 2012; Zhang, 2016) reported the time to cure [SMD = −0.47, 95% CI (−1.56, 0.63)], and the difference in the cure time between the *P. communis* preparations and usual care was not found to be statistically significant. See Figure 11.

One study (Xue, 2012) involving 336 subjects found that *P. communis* preparations significantly reduced the time to resolution of nasal congestion, runny nose, cough, and sneezing.

##### Inflammatory factor outcomes

A study (Li et al., 2022d) involving 100 patients with influenza reported that the *P. communis* preparation was effective in reducing the levels of IL-1, CRP, and TNF-α. After 5 days of treatment, the rate of muscle soreness relief was significantly higher in the treatment group than in the control group.

##### Other outcomes

One study (Hui et al., 2012) of 40 participants with influenza A (H1N1) showed no difference between the two groups in the time to clearance of H1N1 virus.

##### Adverse events

A total of 16 studies (Zhang, 2006; Huai, 2009; Wong et al., 2012; Zhou, 2013; Sun, 2014a; Pan, 2014; Wei, 2014; Cheng et al., 2016; Zhang et al., 2016; Zhang, 2017; Wang, 2020; Ye et al., 2020; Zhou and Qu, 2021; Li et al., 2022a; Li et al., 2022d; Li et al., 2022c) involving 4,194 subjects reported adverse events, of which 6 studies (Sun, 2014b; Cheng et al., 2016; Zhang et al., 2016; Zhang, 2017; Zhou and Qu, 2021; Li et al., 2022a) reported no adverse events in either group, and the remaining 10 studies (Zhang, 2006; Huai, 2009; Wong et al., 2012; Zhou, 2013; Pan, 2014; Wei, 2014; Wang, 2020; Ye et al., 2020; Li et al., 2022c; Li et al., 2022d) had 53 adverse events in the *P. communis* group and 55 in the control group. Statistical analysis revealed no statistically significant differences between the two groups [RR = 0.70, 95% (0.36, 1.35)]. Due to the lack of detailed data in the included studies, it was not possible to differentiate between the types of adverse reactions.

##### Subgroup analysis and sensitivity analysis

After an initial search, we limited the inclusion to people aged 18 years and older. Subgroup analysis of *P. communis* alone and *P. communis* preparations could not be performed because there were no RCTs of *P. communis* as monotherapy. According to the type of control drugs, subgroups were divided into symptomatic treatment and antimicrobial treatment. We did not perform subgroup analyses of antiviral and antibiotic drugs due to limitations in the number of included studies.

We conducted a sensitivity analysis for the primary outcome of high heterogeneity, and the results did not vary. Sample size may be a source of heterogeneity.

##### Publication bias

**FIGURE 12:**
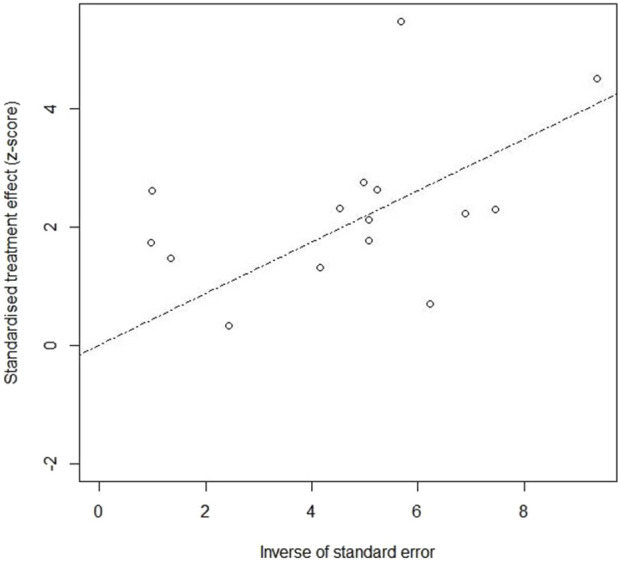
Publication bias in the preparations *versus* usual care.

In the *P. communis versus* usual care, a Galbraith plot (Figure 12) demonstrated that there was symmetry of studies for ARTIs. Publication bias was not present in the linear regression test (Egger’s method). The estimate of bias (intercept) amounted to 1.1753 with a standard error of 0.7066 (p-value 0.1202).

**FIGURE 13:**
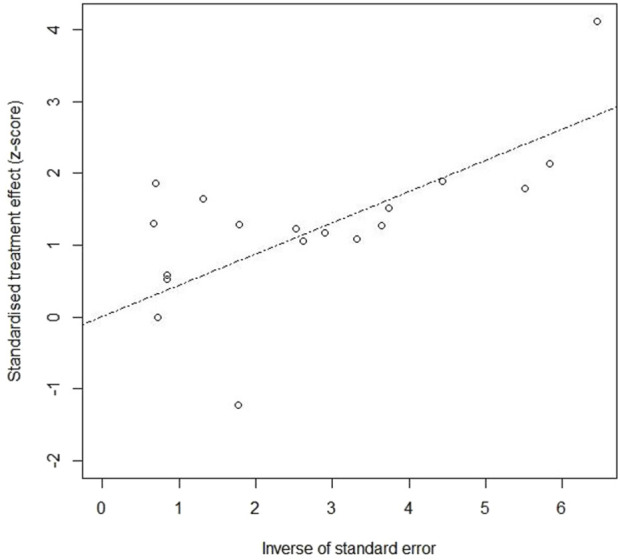
Publication bias in *P. communis* preparations plus usual care *versus* usual care.

In the *P. communis* plus usual care *versus* usual care alone, as shown in Figure 13, the estimate of bias (intercept) amounted to 0.2887 with a standard error of 0.3561 (p-value 0.4294), showing no publication bias in either group.

##### Certainty of evidence

The meta-analysis of four trials comparing *P. communis* preparation with the placebo showed low certainty for the cure rate and low certainty for the fever clearance time in two trials. The meta-analysis of 18 trials comparing *P. communis* preparation combined with usual care with usual care alone showed low certainty for the cure rate and low certainty for the fever clearance time in 4 studies. The meta-analysis of 15 studies comparing *P. communis* preparation with usual care showed very low certainty for the cure rate, very low certainty for the fever clearance time in 4 studies, and very low certainty for the cure time in 3 studies. More information is provided in Supplementary Tables S6–8.

## Discussion

### Summary of the main results

A total of 42 studies involving 6,879 participants were included in the systematic review of *P. communis*, all conducted in China, with 40 published in Chinese and 2 in English. None of the included studies used *P. communis* as monotherapy. Thus, the specific role played by *P. communis* in ARTIs is not clear.

The route of administration for all *P. communis* combinations is oral. *P. communis* preparations improved the cure rate and shortened the time to fever reduction in each of the three control conditions. The combination of *P. communis* preparation with usual care significantly improved lung function and inflammatory response (except for CRP and NEUT%) in patients with AECOPD or acute bronchitis. The *P. communis* preparation significantly increased the clearance rate of influenza virus nucleic acid. However, the number of participants included was too small, and the results may be unstable.

Sixteen studies reported adverse events, and the results showed no statistically significant difference in the incidence of adverse reactions between the *P. communis* group and the control group.

### Quality of the evidence

The methodological quality of the included studies was poor, mainly in terms of non-reporting of the randomization method and allocation concealment in most studies, non-reporting of blinding, lack of sufficient information to judge deviation from the intended intervention, and bias in the measurement of the outcome. Study protocols were found in only two of the included articles, so it is also important to register study protocols before the study and to have a pre-specified analysis plan before starting the analysis. The number of authors for 23 studies was one, and the lack of acknowledgment of other researchers in the text casts doubt on the credibility of the original studies and suggests that multidisciplinary collaborative clinical trials should be conducted in the future. The quality of evidence for all outcomes was assessed using GRADE and was found to be of low or very low certainty.

The studies included have hardly reported the authentication, chemical analysis, and quality control of Chinese herbal medicine, which is detrimental to the reproducibility of experiments. This is because we know that the composition of Chinese herbal medicine may vary depending on the origin, time, and batch of the medicine. Therefore, it is difficult for us to judge the consistency of experimental validity with the herbal compound used.

### Strengths and limitations

This study is the first systematic review to date evaluating *P. communis* for the treatment of ARTIs. We conducted an extensive search in multiple databases and with no language restriction to be as comprehensive as possible.

Studies selected were restricted to adults aged 18 years and older and restricted dosing to oral administration, so we cannot comment on effects in children or alternative preparations. No studies were identified that used *P. communis* as a single herb, and the role played by *P. communis* in the various formulas is not clear, so we are unable to determine if the observed findings are specific to *P. communis*. In addition, Chinese medicine compound formulas consist of a wide range of herbs, among which herbs that are often paired with *P. communis* may have effects similar to those of *P. communis*, which makes the effects of *P. communis* even more difficult to isolate. The included studies were conducted in China, so the conclusions drawn are regionally limited.

As there are not enough studies of identical Chinese medicine compound prescriptions, we combined different compound prescriptions together for analysis. Admittedly, the effects of these herbal compound prescriptions are not identical. In addition, we analyzed different types of ARTIs, such as influenza and cold, acute bronchitis, and AECOPD, which have similar symptoms but are not treated in exactly the same way. It is possible that *P. communis* is more effective for some types of ARTIs than for others.

### Comparison with other studies or reviews

*P. communis* is distributed in several regions, including China, Japan, Korea, Europe, and North America. However, most studies have focused mainly on the role of *P. communis* in the environment, while its medicinal potential remains largely neglected. To our knowledge, there is no published systematic review of *P. communis* for ARTIs. *P. communis* is often used as an assistant medicinal in herbal compound formulas in TCM. Only one formula with *P. communis* as a monarch medicine was included in this review. The results showed that the formula including *P. communis* could alleviate the overall symptoms of ARTIs and shorten the time to fever reduction, which is consistent with the previously published findings of pharmacology experiments (Qian and Jiang, 2014; Zhao et al., 2022) and animal experiments with *P. communis* (Liu et al., 2021; Park et al., 2016; Sun et al., 2016, Liu and Liang, 2014).

### Implications for future research

Although *P. communis* is often used as an assistant medicinal in TCM formulas, this review included a variety of TCM formulas containing *P. communis* for ARTIs, which suggests that *P. communis* plays an important role and is well worth further in-depth study.

A total of 118 herbal medicines were included in this review. The herbs that were most frequently combined with *P. communis* were *Platycodonis Radix* (29 times), *Glycyrrhizae Radix et Rhizoma* (24 times), *Armeniacae Semen Amarum* (19 times), *Forsythiae Fructus* (18 times), *Menthae Haplocalycis Herba* (16 times), and *Scutellariae Radix* (15 times). Future studies may further explore the use of *P. communis* in combination with these herbs to understand how they work together.

The results showed that *P. communis* preparations had significant efficacy in improving symptoms in patients with ARTIs, especially in febrile patients and patients with AECOPD. The results suggest that *P. communis* preparations improve the clearance rate of influenza virus nucleic acid, but due to the small number of participants included, a larger study is recommended to further validate this result in the future. It is also important to identify which preparations are most effective for which types of ARTIs. On the basis of current evidence, this is not possible because there were many preparations and few trials of each. There were no studies that used antibiotic use as an outcome, so future studies could explore whether the use of *P. communis* preparations could reduce antibiotic use.

### Implications for practice

There are currently four Chinese patent medicines containing *P. communis*: Ganmao Qingre granules, antiviral oral liquid, Siji antiviral mixture, Yin Qiao San, and an in-hospital preparation of Qingjie Kanggan granules that can be used directly in the clinical setting. This review found that *P. communis* preparations were effective in alleviating the symptoms of ARTIs and could improve the cure rate. Therefore, *P. communis* preparations have the potential to be used as an alternative symptomatic treatment for ARTIs.

## Conclusion

Low- or very low-certainty evidence demonstrated *P. communis* preparations improve the cure rate, shorten the time to onset of cooling and time to fever reduction, and improve the pulmonary function in ARTIs. Very low-certainty evidence suggests that *P. communis* preparations can improve the inflammatory response caused by ARTIs and increase the clearance rate of influenza virus nucleic acids. No single herb studies were identified, so it is unclear if the observed findings can be attributed to *P. communis*.

However, due to the poor quality of the included studies, these promising findings require further validation. It is recommended to report on the identification of ingredients, quality control, and safety testing of Chinese medicine compounding to increase the reproducibility of the studies. In addition, there were no studies that included the use of antibiotics as an outcome, which could be studied in the future. It is also important to research which combinations of herbs are most effective for which types of ARTIs.

## Data Availability

The original contributions presented in the study are included in the article/Supplementary Material; further inquiries can be directed to the corresponding authors.
