# Supplementary material for: Chinese medicine Phragmites communis (Lu Gen) for acute respiratory tract infections: a systematic review and meta-analysis of randomized controlled trials
Source: Front Pharmacol. 2024 Mar 6;15:1242525. doi: 10.3389/fphar.2024.1242525 (PMC10953292; doi:10.3389/fphar.2024.1242525)
Supplement: Supplementary file 1 [file DataSheet1.docx]

Supplementary Material

Chinese herb *Phragmites communis* (Lu Gen) for acute respiratory tract infections: a systematic review and meta-analysis of randomized clinical trials

Min Fang^1^, Ling-Yao Kong^1^, Guang-He Ji^2^, Feng-Lan Pu^1^, You-Zhu Su^1^, Yu-Fei Li^1^, Michael Moore^3^, Merlin Willcox^3^, Jeanne Trill^3^, Xiao-Yang Hu^3,4*^, Jian-Ping Liu^1*^

^1^Centre for Evidence-Based Chinese Medicine, Beijing University of Chinese Medicine, Beijing, China

^2^School of Traditional Chinese Medicine, Beijing University of Chinese Medicine, Beijing, China

^3^Primary Care and Population Sciences, Faculty of Medicine, University of Southampton, Southampton, United Kingdom

^4^Research Design Service South Central, National Institute of Health Research, Southampton, United Kingdom

*** Correspondence:** Jian-Ping Liu: [Liujp@bucm.edu.cn](mailto:Liujp@bucm.edu.cn;); Xiao-Yang Hu: [X.hu@soton.ac.uk](mailto:X.hu@soton.ac.uk)

# Supplementary Data

Contact the author for data extraction and other relevant data.

# Supplementary Figures and Tables

**Supplementary Table 1 The details of the search for *Phragmites communis* for ARTIs.**

| Databases | Search strategies | Results |
| --- | --- | --- |
| CNKI | **#1** FT=('芦根'+'芦茅根'+'苇根'+'芦头'+'芦柴根'+'顺江龙'+'芦通'+'芦芽根'+'苇子根'+'甜梗子')  **#2** FT=('感冒清热颗粒'+'桑菊感冒片'+'抗病毒口服液'+'复方桑菊感冒片'+'银翘散'+'银翘合剂'+'银翘颗粒'+'桑菊银翘散'+'银翘伤风胶囊'+'清暑解毒颗粒'+'芦根饮'+'桑菊饮')  **#3** SU=('急性呼吸道感染'+'急性鼻咽炎'+'急性上呼吸道感染'+'急性下呼吸道感染'+'普通感冒'+'感冒'+'伤风'+'冒风'+'冒寒'+'急性鼻黏膜炎'+'急性鼻窦炎'+'感染性鼻炎'+'急性鼻炎'+'卡他'+'急性咽炎'+'急性喉炎'+'链球菌性咽炎'+'急性扁桃体炎'+'急性阻塞性喉炎'+'急性会厌炎'+'急性喉咽炎'+'急性疱疹性咽峡炎'+'咽结膜热'+'急性支气管炎'+'急性气管炎'+'急性细支气管炎'+'流感'+'流行性感冒'+'慢性阻塞性肺炎急性发作'+'慢阻肺急性发作'+'COPD急性发作'+'COPD急性加重'+'AECOPD')  **#4** SU=('发热'+'鼻塞'+'流涕'+'咳嗽'+'头痛'+'喉咙痛'+'咽痛'+'咽干'+'咽痒'+'干咳'+'喷嚏'+'急性咽喉痛'+'流鼻涕'+'四肢酸痛')  **#5** (1 OR 2) AND (3 OR 4)  **#6** 5 AND FT % '随机' | 4250 |
| VIP | **#1** U=( 芦根 OR 芦茅根 OR 苇根 OR 芦头 OR 芦柴根 OR 顺江龙 OR 芦通 OR 芦芽根 OR 苇子根 OR 甜梗子 )  **#2** U=(感冒清热颗粒 OR 桑菊感冒片 OR 抗病毒口服液 OR 复方桑菊感冒片 OR 银翘散 OR 银翘合剂 OR 银翘颗粒 OR 桑菊银翘散 OR 银翘伤风胶囊 OR 清暑解毒颗粒 OR 芦根饮 OR 桑菊饮 )  **#3** M=( 急性呼吸道感染 OR 急性鼻咽炎 OR 急性上呼吸道感染 OR 急性下呼吸道感染 OR 普通感冒 OR 感冒 OR 伤风 OR 冒风 OR 冒寒 OR 急性鼻黏膜炎 OR 急性鼻窦炎 OR 感染性鼻炎 OR 急性鼻炎 OR 卡他 OR 急性咽炎 OR 急性喉炎 OR 链球菌性咽炎 OR 急性扁桃体炎 OR 急性阻塞性喉炎 OR 急性会厌炎 OR 急性喉咽炎 OR 急性疱疹性咽峡炎 OR 咽结膜热 OR 急性支气管炎 OR 急性气管炎 OR 急性细支气管炎 OR 流感 OR 流行性感冒 OR 慢性阻塞性肺炎急性发作 OR 慢阻肺急性发作 OR COPD急性发作 OR COPD急性加重 OR AECOPD )  **#4** M=(发热 OR 鼻塞 OR 流涕 OR 咳嗽 OR 头痛 OR 喉咙痛 OR 咽痛 OR 咽干 OR 咽痒 OR 干咳 OR 喷嚏 OR 急性咽喉痛 OR 流鼻涕 OR 四肢酸痛 ）  **#5** (1 OR 2) AND (3 OR 4)  **#6** 5 AND U=随机 | 286 |
| WanFang | **#1** 全部:(芦根 OR 芦茅根 OR 苇根 OR 芦头 OR 芦柴根 OR 顺江龙 OR 芦通 OR 芦芽根 OR 苇子根 OR 甜梗子)  **#2** 全部:(感冒清热颗粒 OR 桑菊感冒片 OR 抗病毒口服液 OR 复方桑菊感冒片 OR 银翘散 OR 银翘合剂 OR 银翘颗粒 OR 桑菊银翘散 OR 银翘伤风胶囊 OR 清暑解毒颗粒 OR 芦根饮 OR 桑菊饮)  **#3** 题名或关键词:(急性呼吸道感染 OR 急性鼻咽炎 OR 急性上呼吸道感染 OR 急性下呼吸道感染 OR 普通感冒 OR 感冒 OR 伤风 OR 冒风 OR 冒寒 OR 急性鼻黏膜炎 OR 急性鼻窦炎 OR 感染性鼻炎 OR 急性鼻炎 OR 卡他 OR 急性咽炎 OR 急性喉炎 OR 链球菌性咽炎 OR 急性扁桃体炎 OR 急性阻塞性喉炎 OR 急性会厌炎 OR 急性喉咽炎 OR 急性疱疹性咽峡炎 OR 咽结膜热 OR 急性支气管炎 OR 急性气管炎 OR 急性细支气管炎 OR 流感 OR 流行性感冒 OR 慢性阻塞性肺炎急性发作 OR 慢阻肺急性发作 OR COPD急性发作 OR COPD急性加重 OR AECOPD)  **#4** 题名或关键词:(发热 OR 鼻塞 OR 流涕 OR 咳嗽 OR 头痛 OR 喉咙痛 OR 咽痛 OR 咽干 OR 咽痒 OR 干咳 OR 喷嚏 OR 急性咽喉痛 OR 流鼻涕 OR 四肢酸痛)  **#5** (1 OR 2) AND (3 OR 4)  **#6** 5and 全部:(随机) | 331 |
| CBM | **#1** ("感冒清热颗粒"[常用字段:智能] OR "桑菊感冒片"[常用字段:智能] OR "抗病毒口服液"[常用字段:智能] OR "复方桑菊感冒片"[常用字段:智能] OR "银翘散"[常用字段:智能] OR "银翘合剂"[常用字段:智能] OR "银翘颗粒"[常用字段:智能] OR "桑菊银翘散"[常用字段:智能] OR "银翘伤风胶囊"[常用字段:智能]) OR (("清暑解毒颗粒"[常用字段:智能] OR "芦根饮"[常用字段:智能] OR "桑菊饮"[常用字段:智能]) OR ("甜梗子"[全部字段:智能]) OR ("芦根"[全部字段:智能] OR "芦茅根"[全部字段:智能] OR "苇根"[常用字段:智能] OR "芦头"[常用字段:智能] OR "芦柴根"[常用字段:智能] OR "顺江龙"[常用字段:智能] OR "芦通"[常用字段:智能] OR "芦芽根"[常用字段:智能] OR "苇子根"[常用字段:智能]))  **#2** ("流鼻涕"[常用字段:智能] OR "四肢酸痛"[常用字段:智能]) OR ("咳嗽"[常用字段:智能] OR "头痛"[常用字段:智能] OR "喉咙痛"[常用字段:智能] OR "咽痛"[常用字段:智能] OR "咽干"[常用字段:智能] OR "咽痒"[常用字段:智能] OR "干咳"[常用字段:智能] OR "喷嚏"[常用字段:智能] OR "急性咽喉痛"[常用字段:智能]) OR ("流行性感冒"[常用字段:智能] OR "慢性阻塞性肺炎急性发作"[常用字段:智能] OR "慢阻肺急性发作"[常用字段:智能] OR "COPD急性发作"[常用字段:智能] OR "COPD急性加重"[常用字段:智能] OR "AECOPD"[常用字段:智能] OR "发热"[常用字段:智能] OR "鼻塞"[常用字段:智能] OR "流涕"[常用字段:智能]) OR ("急性阻塞性喉炎"[常用字段:智能] OR "急性会厌炎"[常用字段:智能] OR "急性喉咽炎"[常用字段:智能] OR "急性疱疹性咽峡炎"[常用字段:智能] OR "咽结膜热"[常用字段:智能] OR "急性支气管炎"[常用字段:智能] OR "急性气管炎"[常用字段:智能] OR "急性细支气管炎"[常用字段:智能] OR "流感"[常用字段:智能]) OR ("急性鼻黏膜炎"[常用字段:智能] OR "急性鼻窦炎"[常用字段:智能] OR "感染性鼻炎"[常用字段:智能] OR "急性鼻炎"[常用字段:智能] OR "卡他"[常用字段:智能] OR "急性咽炎"[常用字段:智能] OR "急性喉炎"[常用字段:智能] OR "链球菌性咽炎"[常用字段:智能] OR "急性扁桃体炎"[常用字段:智能]) OR ("急性呼吸道感染"[常用字段:智能] OR "急性鼻咽炎"[常用字段:智能] OR "急性上呼吸道感染"[常用字段:智能] OR "急性下呼吸道感染"[常用字段:智能] OR "普通感冒"[常用字段:智能] OR "感冒"[常用字段:智能] OR "伤风"[常用字段:智能] OR "冒风"[常用字段:智能] OR "冒寒"[常用字段:智能])  **#3** "随机"[全部字段:智能]  **#4** 1 AND 2 AND 3 | 422 |
| PubMed | **#1** rhizoma phragmites OR reed rhizome OR phragmites communis  OR common reed rhizome OR lu gen OR Yinqiaosan OR yinqiao san OR yin-qiao-san OR powder of Lonicera and Forsythia OR Yinqiao powder OR lonicerae and forsythiae powder  **#2** Random*[title/abstract]  **#3** 1 AND 2 | 12 |
| Embase | **#1** 'rhizoma phragmites' OR (('rhizoma'/exp OR rhizoma) AND ('phragmites'/exp OR phragmites)) OR (reed AND rhizome) OR 'phragmites communis' OR (common AND reed AND rhizome) OR 'lu gen':ti,ab,kw OR yinqiaosan OR (yinqiao AND san) OR 'yin qiao san' OR (powder AND of AND lonicera AND forsythia) OR (yinqiao AND powder) OR (lonicerae AND forsythiae AND powder)  **#2** random*  **#3** 1 AND 2 | 21 |
| Web of Science | **#1** ALL=(rhizoma phragmites or reed rhizome or phragmites communis or common reed rhizome or Yinqiaosan OR yinqiao san OR yin-qiao-san OR powder of Lonicera and Forsythia OR Yinqiao powder OR lonicerae and forsythiae powder) | 23 |
| Cochrane Library | **#1** (rhizoma phragmites or reed rhizome or phragmites communis or common reed rhizome)" (Word variations have been searched)  **#2** (Yinqiaosan) OR (yinqiao san) OR (yin-qiao-san) OR (powder of Lonicera and Forsythia) OR (Yinqiao powder) OR (lonicerae and forsythiae powder)  **#3** (Random*):ti,ab,kw  **#4** 1 OR 2  **#5** 3 AND 4 | 18 |
| Chinese Clinical Trial Registry | **#1** 干预： 感冒清热颗粒 OR 桑菊感冒片 OR 抗病毒口服液 OR 复方桑菊感冒片 OR 银翘散 OR 银翘合剂 OR 银翘颗粒 OR 桑菊银翘散 OR 银翘伤风胶囊 OR 清暑解毒颗粒 OR 芦根饮 OR 桑菊饮  **#2** 疾病：急性呼吸道感染 OR 急性鼻咽炎 OR 急性上呼吸道感染 OR 急性下呼吸道感染 OR 普通感冒 OR 感冒 OR 伤风 OR 冒风 OR 冒寒 OR 急性鼻黏膜炎 OR 急性鼻窦炎 OR 感染性鼻炎 OR 急性鼻炎 OR 卡他 OR 急性咽炎 OR 急性喉炎 OR 链球菌性咽炎 OR 急性扁桃体炎 OR 急性阻塞性喉炎 OR 急性会厌炎 OR 急性喉咽炎 OR 急性疱疹性咽峡炎 OR 咽结膜热 OR 急性支气管炎 OR 急性气管炎 OR 急性细支气管炎 OR 流感 OR 流行性感冒 OR 慢性阻塞性肺炎急性发作 OR 慢阻肺急性发作 OR COPD急性发作 OR COPD急性加重 OR AECOPD | 177 |
| Clinicaltrial.gov | **#1** Acute nasopharyngitis common cold OR Acute sinusitis OR Acute pharyngitis OR Acute tonsillitis OR Acute laryngitis and tracheitis OR Acute obstructive laryngitis and epiglottitis  **#2** Influenza OR AECOPD OR Acute exacerbation chronic obstructive pulmonary disease OR Acute exacerbation of COPD OR Acute bronchitis OR Acute bronchiolitis  **#3** Acute respiratory tract infection OR ARTI OR Acute respiratory infections OR Acute upper respiratory tract infection OR Acute lower respiratory tract infection | 3280 |

**Supplementary Table 2 Characteristics of included randomized trials**

| **ID** | **Diagnosis** | **Setting** | **Duration symptoms** | **Age**  **(year)** | **Analysis size** | **Sample size**  **（T/C）** | **Gender(male)**  **(T/C)** | **Intervention group** | **Control group** | **Control group details** | **Outcomes** | **During treatment** |
| --- | --- | --- | --- | --- | --- | --- | --- | --- | --- | --- | --- | --- |
| Huai W 2009 | AURTIs | China | Within 2 days | 18-58 | 86/86 | 43/43 | NR | Ganmao Qingre Keli | Symptomatic treatment | Pseudoephedrine oral solution (100 ml per bottle, each 10 ml contains 30mg of pseudoephedrine hydrochloride, 10mg of dextromethorphan hydroxide, and 2mg of paracetamol), 10 ml per dose, 3 times daily. | 5,6,7,8,33 | NR |
| Wang QM 2010 | AURTIs | China | Within 3 days | 18-33/18-29 | 160/160 | 100/60 | 78/32 | Yinqiao San + control group | Antivirals + antibacterial therapy | 5% dextrose 250ml+viriconazole 0.4g+ceftriaxone sodium 2.0g, IV, once a day | 1,3,4 | 5d |
| Zhang XT 2016 | Wind-heat cold | China | NR | 42.3 (18-61)/41.9 (19-62) | 124/124 | 61/63 | 31/31 | Yinqiao San | Symptomatic treatment | Compound Aminophenalkylamine Gum Huan (trade name: Quick, Zhejiang Yafeng Pharmaceutical Co., Ltd. production, product batch No. 141101, the composition of acetaminophen, amantadine hydrochloride, chlorpheniramine maleate. Artificial cowberry. Caffeine). 1 capsule/time. 2 times / d. Double the dose for severe hyperthermia | 1,2,3,4 | 3d |
| Zhang MX 2006 | AURTIs | China Outpatient and Inpatient | Within 48 hours | 41.69±15.82(18-65)/40.68±16.18(18-65) | 300/300 | 160/140 | 86/68 | Self-designed formula for clearing heat, detoxifying, and nourishing Yin | Symptomatic treatment + antivirals + antibacterial treatment | Aspirin, antibiotics, antivirals (morpholinoformin hydrochloride or aciclovir) as appropriate | 1,3,4 | Possible 3d |
| Xu XF 2013 | AURTIs | China | 1-2 days | 31.10 (20-43)/31.50 (19-44) | 70/70 | 35/35 | 16/17 | Anti-fever formula | Symptomatic treatment | Phenergan (0.5 g/65 mgx10 tablets, Sino-US Shanghai Schweppes Pharmaceutical Co., Ltd.) Oral treatment, 1 tablet per dose every 6 hours. | 1 | 36h |
| Sun JF-1 2014 | AURTIs | China | Within 48 hours | 30.24±2.01/28.36±1.94 | 38/NR | 19/19 | 10/9 | Sangju Yinqiao Combination | Symptomatic treatment | Meprobamate Tablets (Sino-US Tianjin Shiq Pharmaceutical, Production Lot No. 12110394):l# po q12h | 1,4,12,33 | 5d |
| Sun JF-2 2014 | AURTIs | China | Within 48 hours | 30.21±1.74/32.47±0.89 | 37/NR | 18/19 | 10/11 | Qingre Huashi Oral Liquid | Symptomatic treatment | Meprobamate Tablets (Sino-US Tianjin Shiq Pharmaceutical, Production Lot No. 12110394): 1# po q12h | 1,4,12,33 | 5d |
| Song DS 2021 | AECOPD | Hospitalization in China | NR | 68.79±9.23 (56-89)/67.79±8.14 (55-90) | 96/96 | 48/48 | 27/29 | Pus-removal and detoxification soup + control group intervention | Antimicrobial therapy + anti-inflammatory therapy + supportive therapy | (1) Levofloxacin hydrochloride injection (manufactured by Shiyao Yinhu Pharmaceutical Co., Ltd., lot no. 20171205, 100 mL:0.3 g), 100 mL each time, intravenous infusion, once every 12 hours; (2) Budesonide suspension for inhalation (manufactured by Zhengda Tianqing Pharmaceutical Co., Ltd., lot no. H20203063, 2 mL each), mixed with 2 mL of physiological saline and then nebulized, twice a day; (3) Keep the airway unobstructed, continuous low-flow oxygenation, supplementation of water and salt to correct electrolyte disorders, glucose, vitamins and other nutritional support treatment, etc. (3) keep the airway open, continuous low-flow oxygenation, supplementation of water and salt to correct electrolyte disorders, glucose, vitamins and other nutritional support treatment, etc. | 1,14,15,18,22 | 4w |
| Xue XF 2012 | AURTIs | China Outpatient | Within 3 days | 50.1±14.4(18-65)/48.9±14.6(18-65) | 336/336 | 168/168 | 82/89 | Kangbingdu Oral Liquid | Antiviral therapy | Ribavirin tablets 0.15g, po, tid | 2,4,5,6,7,11 | NR, possibly 1-4d |
| Sun L 2022 | ARTIs | Hospitalization in China | Within 48 hours | 44.37±20.70(18-70)/45.43±18.20(19-70) | 60/60 | 30/30 | 13/15 | Antipyretic pellets + control group treatment | Antiviral therapy + anti-infective therapy + cooling therapy + conventional supportive therapy | (1) antiviral treatment: patients with influenza were given oseltamivir capsules orally; (2) anti-infective treatment: for bacterial infections, levofloxacin was used for initial empirical treatment, and cefoperazone sodium sulbactam clinical study 20 sodium was used for anti-infective treatment if necessary, and sensitive antibiotics were used for those with drug sensitivity test results; (3) cooling treatment: for patients with body temperature greater than 38.5℃, physical cooling treatment was preferred. If the body temperature is greater than 38.5℃ and does not subside for 4 hours, indomethacin suppositories should be given to reduce the anal fever, and the clinical study should be terminated after the judgment of the attending physician; ③ Routinely give rehydration treatment, and advise patients to avoid wind and cold, drink more water, diligently ventilate, eat lightly, pay attention to rest, and avoid smoking and alcohol during the treatment period. | 1,3,4 |  |
| Li YP 2022 | influenza | China | Within 48 hours | 36.9±7.65/36.20±7.99 | 100/100 | 50/50 | 28/31 | Chailing Shigao soup | Antiviral therapy | Oseltamivir phosphate capsule (Yichang Changjiang Pharmaceutical Co., Ltd., State Drug Administration: H20065415) 75 mg, 1 time daily orally. | 1,4,5,67,9,10,12,18,21,26,33 | 5d |
| Zhou ZW 2021 | Acute pharyngitis | China Outpatient | 1-10 days/1-9days | 56.25±9.21(47-89)/55.95±8.22(48-86) | 80/80 | 40/40 | 0 | Self-designed Nourishing Yin and Clearing Liver Soup with Addition and Reduction | Symptomatic treatment | Compound Aminophenalkylamine Tablets (manufactured by Jilin Wu Tai Sensitive Health Pharmaceutical Co., Ltd., State Drug Administration: H22026193) | 1,33 | 5d |
| Li J 2020 | AECOPD | Hospitalization in China | NR | 64.90±5.98/65.57±6.17 | 60/60 | 30/30 | 17/19 | Ma Gan Cough Relief and Asthma Calming Soup + Control group treatment | Anti-inflammatory treatment + antibacterial treatment + symptomatic treatment + supportive treatment | The control group was treated with conventional Western medicine (refer to Global Strategy for the Diagnosis, Treatment, and Prevention of Chronic Obstructive Pulmonary Disease 2019: low-flow oxygenation, anti-inflammatory (adjust antibiotics according to sputum culture and drug sensitivity results: apply hormones if necessary). Asthma (Diprophylline (asthma) Injection-Tianjin Jinyao Pharmaceutical Co., Ltd., Approval No. H12020958), phlegm (ambroxol Hydrochloride Injection-Shanghai Boehringer Ingelheim Pharmaceutical Co. | 1,12,13,15,16,26,29 | 14d |
| Luo HL 2019 | Acute cough | China Outpatient | Within 3 weeks | 48.25±6.84(18-70)/47.74±6.37(18-70) | 120/120 | 60/60 | 20/18 | Self-formulated Chinese medicine | Antibacterial treatment + symptomatic treatment | Treatment by conventional Western medicine, such as antibiotics, cough suppressants, etc. | 1 | 6d |
| Hu QY 2019 | Acute sinusitis | China | Within 5 days | 35.86 (20-55)/36.06 (18-53) | 107/107 | 56/51 | 32/21 | Adding Flavor to Qianjin Reed Stem Soup | Antimicrobial therapy | Amoxicillin Clavulanic Acid Potassium Dispersible Tablets (manufactured by HaPharma Group Pharmaceutical General Factory, approval number: State Drug Administration H20041621), 457 mg per dose, 3 times daily. | 1 | 7d |
| Yang LY 2019 | AURTIs | China | Within 48 hours | 38.26±2.36/38.26±2.36 | 100/100 | 50/50 | 25/25 | Waigan Qingre jiedu formula + placebo of phenolamine curare tablets | Placebo | Placebo and Pheniracetam Placebo contain the ingredients of Externally Induced Fever Relief Formula at a dose of 1/40, manufactured by Shenzhen Sanjiu Pharmaceutical Company. The manufacturer of the phenolamine curtain placebo is Suzhou Yusen New Drug Development Co. | 1,3,4 | 2-3d |
| Xue JH 2017 | Common cold fever | China Outpatient | Within 48 hours | 36.7±6.7/39.4±2.1 | 96/96 | 48/48 | 25/20 | Waigan Qingre jiedu formula formula + paracetamol tablets placebo | Placebo | paracetamol Tablet (Shanghai Xinyi Pharmaceutical Co., Ltd., State Drug Licence H31022508) 1 tablet, to be taken orally 3 times a day. At the same time, the placebo of External Sense Heat and Toxin Relief Agreement Formula was given, provided by China Resources Sanjiu Pharmaceutical Co., Ltd. The 1/40 dosages of each medicine of External Sense Heat and Toxin Relief Agreement Formula were mixed and made into 1 sachet uniformly, with the same appearance as that of External Sense Heat and Toxin Relief Agreement Formula. Take 1 sachet with boiling water 3 times daily. | 1 | 3d |
| Zhang ZX 2017 | AECOPD | Hospitalization in China | NR | 67.03±4.56/64.87±5.08 | 62/62 | 31/31 | 23/20 | Waigan Qingre jiedu formula + control group treatment | Antibacterial treatment + symptomatic treatment + supportive treatment | According to the conventional treatment and care, sensitive antimicrobial drugs were selected according to the condition, sputum culture + drug sensitivity test results (before obtaining sputum culture + drug sensitivity test results, antimicrobial drugs were selected based on experience), and albuterol 1ml + ipratropium bromide 2m1 + saline 2m1, nebulized inhalation (3 times/day) to relieve spasm and asthma, aminoglutethimide hydrochloride extended-release capsule (75mg/time, 1 time/day) to expectorate, controlled oxygen therapy (to maintain oxygen saturation at 88%) to improve hypoxia and other symptomatic supportive treatments. (75mg/time, 1 time/day), sputum expectoration, controlled oxygen therapy (to maintain oxygen saturation above 88%) to improve hypoxia, and other symptomatic support treatment. | 1,12,14,15,16,17,26,27,28,29,30 | 14d |
| Cheng Y 2016 | AURTIs | China | Within 48 hours | 36.79±13.67/37.21±15.43 | 81/ 96 | 41/40 | 22/20 | Waigan Qingre jiedu formula + placebo of phenolamine curare tablets | Placebo | Placebo and Pheniracetam Placebo contain the ingredients of Externally Induced Fever Relief Formula at a dose of 1/40, manufactured by Shenzhen Sanjiu Pharmaceutical Company. The manufacturer of the phenolamine curtain placebo is Suzhou Yusen New Drug Development Co. | 1,3,4,33 | 3d |
| Sun YT 2014 | influenza | China Outpatient | Within 48 hours | 32.5±9.84 (18-53)/32.9±9.65 (19-55) | 58/60 | 30/28 | 11/11 | Qingwen Baidu formula pellets + control group treatment | Symptomatic treatment | Ibuprofen Extended Release Capsules 0.3g/dose, if necessary, manufactured by Sino-American Tianjin Shih-Ke Pharmaceutical Co. | 1,12,4,32,33 | 5d |
| Wei ZJ 2014 | AECOPD | Hospitalization in China | 2-24 days/3-17 days | 67.00±6.18(45-74)/68.21±5.68(48-75) | 67/72 | 33/34 | 19/22 | Clearing heat and detoxifying Fu Zheng Tang + control group treatment | Antibacterial treatment + symptomatic treatment + supportive treatment | According to conventional treatment and care, sensitive antimicrobial drugs were selected according to the condition and sputum culture + drug sensitivity test results (before obtaining sputum culture + drug sensitivity test results, antimicrobial drugs were selected based on experience), and symptomatic supportive treatment such as antispasmodic and asthma, cough suppression, sputum reduction, controlled oxygen therapy, and maintenance of water/electrolyte and acid-base balance was given. | 1,12,14,15,16,17,28,29,30,33 | 14d |
| Pan XS 2014 | AURTIs | China Outpatient | Within 48 hours | 18-20 | 40/40 | 40/40 | 13/15 | Four Seasons Anti-Viral Combination | Antiviral therapy | Ribavirin tablets are taken orally, 30 mg/dose, 3 times daily, for 3 d | 1,33 | 3d |
| Zhou JC 2013 | AURTIs | China | Within 36 hours | 41.20±14.25/39.67±14.82 | 159/159 | 80/79 | 29/27 | Anti-sensitive granules | Placebo | Anti-sensitive granules, 4g/bag, provided by Wuhu Green Leaf Pharmaceutical Co. | 1,12,33 | NR, possibly 3d |
| Wang FW 2007 | influenza | China | 2 hours-7 days/3 hours-6 days | 18-23/18-24 | 220/220 | 120/100 | 98/82 | Clearing heat and detoxifying the throat | Antiviral therapy + antibacterial therapy + supportive therapy | 5% dextrose 250 ml + ribavirin 0.6-0.8 g + leucomycin 1 million U, intravenous infusion, once a day | 1 | 5d |
| Li CY 2022 | AECOPD | China | 4-38 hours/5-40 hours | 50.62±4.48(40-73)/51.29±4.62(41-74) | 104/104 | 52/52 | 27/29 | Ma Heng Di Bai San plus reduction + control group treatment | Antibacterial treatment + symptomatic treatment | All patients were treated with Western medicine alone and were given antipyretic, phlegm-suppressing, cough-suppressing, asthma-suppressing, bronchodilating, and empirical anti-infective therapy upon admission. Isoproterenol (Boehringer Ingelheim Pharmaceuticals, specification: 2 m L/stem, batch numbers: 20170403, 20180904, 20191003, 20200304) 2 m L + budesonide inhalation (Astra Zeneca Pty Ltd, specification: 1 mg/stem, batch numbers: 20170406, 20180705, 20190304 The inhalation of Budesonide (Astra Zeneca Pty Ltd, specification: 1 mg/stem, lot no. 20170406, 20180705, 20190304, 20200502) 1 mg, 3 times/d. Cephalexin sulbactam for injection (trade name: Shu Shen, Pfizer, specification: 0.3 g/stem, lot no. 20170602, 201180703, 20181203, 20190904) 2 g, 12 h drip. | 1,12,13,14,15,23,26 | 2w |
| Li HY 2022 | AECOPD | China | NR | 75.30±8.11 (60-85)/74.63±6.12 (61-85) | 63/66 | 33/30 | 31/28 | Beneficial Qi, nourishing Yin, pungent and dispersing phlegm formula + control group treatment | Antibacterial treatment + symptomatic treatment + supportive treatment | Western medicine conventional treatment. Specifically:1) Anti-infective drugs. Piperacillin tazobactam sodium for injection (Hunan Zhongnan Kellen Pharmaceutical Co., Ltd., Approval No.: State Drug Quorum H20074053, Specification: 2.25 g/bottle) 4.5 g diluted with 0.9% sodium chloride injection 50 ~ 100 ml, intravenous drip, 8~12 h once, 5~10 d for a course, 2) bronchodilators. Budesonide suspension for inhalation (Health Element Pharmaceutical Group Co., Ltd., Approval No.: State Drug Administration H20203343, Specification: 2 ml:1 mg), nebulized inhalation, 2 times/d, 3~5 d as a course of treatment: 3) Expectorants. Ltd., Approval No.: State Drug Administration H20133026, Specification: 2 ml: 15 mg) 30 mg diluted with 0.9% sodium chloride injection 50 ~ 100 ml, intravenous drip, 12 h for 1 time, 3~5 d for 1 course: acetylcysteine solution for inhalation (ZAMBON S.p.A imported drug) Registration No. H20150548, specification: 3 ml:0.3 g) 0.3 mg, nebulized inhalation, 2 times/d, 5~10 d for 1 course: 4) low flow oxygen. 12~15 h/d, 10 d for 1 course, non-invasive assisted ventilation, and other symptomatic treatment as needed. | 12,13 | 10d |
| Zheng YL 2019 | AECOPD | China | NR | 66.13±8.16(52-76)/67.91±8.35(54-78) | 70/70 | 35/35 | 21/21 | Qing lung and phlegm decoction + control group treatment | Anti-infection + symptomatic treatment + supportive treatment | According to the actual situation, routine anti-infection, antispasmodic and asthma, sputum low-flow oxygen, and other conventional drugs are given to treat the symptoms. | 1 | 7d |
| Zhang ZX 2016 | AECOPD | China | NR | 66.22±7.32/66.78±4.48 | 62/62 | 31/31 | 26/23 | Clearing heat and detoxifying Fu Zheng Tang + control group treatment | Antibacterial treatment + symptomatic treatment + supportive treatment | The patient was treated with sensitive antimicrobial drugs according to the condition and the results of sputum culture + drug sensitivity test (before the results of sputum culture + drug sensitivity test were obtained, BESTEC (cephalexin hydrochloride for injection, 1 g/dose, 2 times/day) was used) and was given salbutamol (200 μg/dose, less than 8 times a day) to relieve spasm and asthma, ambroxol hydrochloride tablets (2 tablets/dose, 3 times/day) to remove sputum, controlled oxygen therapy (oxygen flow rate of 2 L/min) to improve hypoxia, and to maintain water/electrolyte and acid-base balance. (2 L/min) to improve hypoxia, maintain water/electrolyte and acid-base balance, and other symptomatic support treatments. | 1,12,14,15,16,17,33 | 14d |
| Chen H 2014 | AECOPD | China | 8-25 days/7-23 days | 73.25(50-78)/75.16(52-82) | 60/60 | 30/30 | 19/18 | Clear heat and resolve phlegm formula + control group treatment | Anti-infection + symptomatic treatment + supportive treatment | Oxygen therapy, anti-infection, expectoration, aminophylline. | 1,16 | NR |
| Hui P 2012 | Influenza A (H1N1) | China | NR | 18-47/20-49 | 40/40 | 20/20 | 11/10 | Self-designed purging and detoxifying soup | Antiviral therapy | Oral oseltamivir is administered according to the recommended dosing regimen of the Ministry of Health, i.e., 75 mg twice daily for adults. | 1,4,32 | 5d |
| Shi JP 2010 | AECOPD | China | Within 14 days | 72.6±7.8 (52-83) | 40/40 | 20/20 | NR | Spleen Strengthening, Lung Clearing, Blood Invigorating Formula + Control Group Treatment | Antibacterial treatment + symptomatic treatment + supportive treatment | Select therapeutic drugs according to the severity of the disease  The treatment drugs, such as controlled oxygen therapy, antibiotics, bronchodilators, glucocorticoid  glucagon, non-invasive mechanical ventilation, etc. | 1,14 | 10d |
| Wong 2012 | AURTIs | China | Within 48 hours | 44.3±10.4 (20-71)/44.3±11.5 (18-74) | 165/165 | 82/83 | 44/40 | Silver Chiao San | Placebo | The CHM placebo was supplied by a local pharmaceutical company that manufactures these two CHM formulae in the form of concentrated granules for sale. These granules are extracted from the Chinese herbs of the specific formulae replicating the traditional method of preparing decoction using modern extraction and concentration technologies, according to the Good Manufacturing Practice. These granules are extracted from the Chinese herbs of the specific formulae replicating the traditional method of preparing decoction using modern extraction and concentration technologies, according to the Good Manufacturing Practice (GMP)standards. All granules were chemically standardized,  All granules were chemically standardized, and granule size and weight in each sachet were carefully  Dosing instructions were based on the recommendations of the manufacturer (Two 7-g Dosing instructions were based on the recommendations of the manufacturer (Two 7-g sachets two times daily). | 31,33 | Within 10d |
| Hong LL 2020 | AECOPD | China | NR | 61.02±3.13(50-75)/60.71±3.29(50-75) | 86/86 | 43/43 | 22/20 | Tiger lutefisk + control group treatment | Anti-infection + symptomatic treatment + supportive treatment | The control group was given oxygen therapy, bronchodilators, nutritional support, expectoration and cough suppression, infection control, maintenance of water-electrolyte acid-base balance, and symptomatic support. | 1,14,15,17,21,29,20,31 | 2w |
| Wang DY 2020 | AECOPD | China | NR | 74.32±6.94(66-82)/75.12±7.51(65-84) | 80/80 | 40/40 | 23/25 | Clear lung and resolve phlegm soup + control group treatment | Antibacterial treatment + symptomatic treatment + supportive treatment | Western medicine conventional treatment: ①Injectable sodium ceftriaxone (Shanghai Huayuan Anhui Renji Pharmaceutical Co., Ltd., production batch No. 16121132, specification: 1g/branch) 2g, intravenous infusion, 1 time/d: ②Oral administration of amiloride oral solution (Sunstone (Tangshan) Pharmaceutical Co., Ltd., production batch No. 16040752, specification: 10 mL:30 mg) 30 mg/d, 2 times /③Nasal catheter oxygen (4L/min); ④Other symptomatic treatment | 4,7,8,33 | 13d |
| Ye D 2020 | Acute bronchitis | China | NR | 39.25±8.34(19-75)/39.41±8.37(18-75) | 64/64 | 32/32 | 20/19 | Treatment with Qing-Heat and Phlegm Removal Tang Plus + Control Group | Anti-infection treatment + symptomatic treatment | Conventional Western medicine treatment. Piperacillin sodium sulbactam for injection (Hubei Weil Pharmaceutical Co., Ltd., State Drug Quantifier H20051606, specification: 1.5 g), intravenous infusion, 3 g/d, 2 times/d; compound methocarbamol capsule (Changxing Pharmaceutical Co., Ltd., State Drug Quantifier H20020393, specification: 46.5 mg x 48 s), 2 s/d, 3 times/d; carbocysteineCarbocysteinearbocysteine oral solution (Beijing Chengji Pharmaceutical Co. (Beijing Chengji Pharmaceutical Co., Ltd., State Drug Quantifier H20044967, Specification: 10mLx10pcs), 10 ml/dose, 3 times/d. | 1,25,26,33 | 7d |
| Bi DX 2019 | Acute bronchitis | Hospitalization in China | 3-12 days | 48.78±6.23(31-63)/48.78±6.23(31-63) | 84/84 | 42/42 | 28/28 | Clear heat, promote lung and stop cough soup + control group treatment | Anti-infective treatment + symptomatic treatment + supportive treatment | Patients in the control group were treated with symptomatic interventions in Western medicine, including cough suppression, expectoration, cooling, nutritional support, and correction of internal environmental disorders; and targeted anti-infection according to the type of infection, including intravenous cefoperazone sulbactam (Sinopharm Group Weichida Pharmaceutical Co., Ltd., specification 3.0 g, production batch No. 20163314) 1 g/time, mixed with 0.9% sodium chloride injection 150 For virus infection, intravenous ribavirin (Yangtze River Pharmaceutical Group Nanjing Hailing Pharmaceutical Co., Ltd., specification 0.5 g, production batch No. 20160314B) 0.3 g/time, mixed into 0.9% sodium chloride injection 150 mL, twice a day. | 1,18,24,28,29,30 | 7d |
| Guo XH 2009 | Bacterial tonsillitis | China | 1-3 days | 18-64 | 100/100 | 60/40 | NR | Yin Qiao San plus reduction | Symptomatic treatment | Oral Neocontrol capsules 1 capsule twice a day | 1 | 3d |
| Zong Y 2020 | Influenza | China Outpatient | Within 48 hours | 35.2±5.9 (19-54)/33.5±10.1 (18-51) | 120/120 | 60/60 | 32/29 | Self-formulated anti-influenza formula + control group treatment | Antiviral therapy | Oseltamivir capsules (Yichang Changjiang Pharmaceutical Co., Ltd., GMP H20065415, Specification 75 mg/capsule, Lot No.: 180235) treatment, 1 capsule each time, 2 times daily. | 1,3,4,32 | 3d |
| Chen XM 2016 | AURTIs | Hospitalization in China | 2-23 days | 38.75±2.03(20-88)/39.02±2.14(21-88) | 200/200 | 100/100 | 48/53 | Dredging Wind and Promoting Lung Soup with Addition | Antimicrobial therapy | The patient was treated with conventional Western medicine and given amoxicillin dispersible tablets (Shanxi Tongda Pharmaceutical Co., Ltd., State Drug Administration H20000493), 0.5g/time, 3 times/d. | 1 | 5d |
| Guo XX 2017 | Acute pharyngitis | China Outpatient | NR | 37.2 (20-70)/37.6 (21-72) | 63/63 | 33/30 | 18/14 | Clearing the throat with black bean curd soup | Antimicrobial therapy | Amoxicillin capsule (produced by Hainan Sanye Wonderful Pharmaceutical Co., Ltd., State Pharmacopoeia: H46020281) treatment, 0.5 g per time, 3 times daily. | 1,12 | 7d |
| Chang QJ 2016 | AECOPD | Hospitalization in China | NR | 68.6±5.5 (51-80)/68.4±5.9 (52-81) | 84/84 | 42/42 | 26/25 | Xuanbai Chengqi Tang + control group treatment | Antibacterial treatment + symptomatic treatment + supportive treatment | The control group was given conventional Western medical treatment: bed rest, 0.4 g moxifloxacin or 0.6 g levofloxacin intravenously (both 1 time/d), or antibiotics according to the sputum culture results: 0.3 g doxorubicin (1 time/d) and 30 mg amiloride hydrochloride (2 times/d) per drip, and low-flow oxygenation. | 1,14,15 | 14d |
| Xu GJ 2014 | AURTIs | Hospitalization in China | 5.7±2.3 days/5.8±2.2 days | 35/36 | 90/90 | 45/45 | 23/25 | Silver wattle and willow antipyretic soup + control group treatment | Anti-infection treatment | The control group was given penicillin 8 million U/d, with virazole 0.3 g/d intravenously for 3~5 d, and supplemented with combination therapy. | 1,3,4 | 3d |
| Li HJ 2022 | Influenza, common cold | China | Within 48 hours | 36.1±14.9/36.1±14.81 | 2647/2647 | 1986/661 | 1086/358 | Anti-viral Oral Liquid | Placebo | Improvement rate of clinical symptoms, time to resolution of fever, time to onset of cooling, recovery rate, adverse reactions | 33 | 5d |

Note:1cure rate, 2cure time, 3cooling onset time, 4Fever clearance time, 5Nasal congestion relief rate or disappearance time, 6Rate of relief or time to disappearance of runny nose, 7Cough relief rate or time to disappearance, 8Rate or time to resolution of cough sputum, 9Sore throat relief rate or disappearance time, 10Rate of muscle soreness relief or time to disappearance, 11Sneeze relief rate or disappearance time, 12TCM Syndrome scores, 13CAT scores, 14FEV1, 15FEV1/FVC, 16FEV1%, 17FVC, 18TNF-α, 19TGF-β, 20GRO-α, 21 IL-10, 22IL-8, 23IL-6, 24IL-4, 25PCT 26 CRP, 27 SAA, 28 WBC, 29NEUT%, 30LYM%, 31quality of life, 32Negative rate of influenza virus nucleic acid

**Supplementary Table 3 Details of Chinese patent medicines included in the trials.**

| **ID** | **Name** | **Diagnosis（syndrome differentiation）** | **How to take the medication？** | **Reference to publicly available materials, such as pharmacopeia, for the details about the composition, dosage, efficacy, safety** | **Details of the formula, namely 1) the proprietary product name (i.e., brand name), 2) name of manufacturer, 3) lot number, 4) production date and expiry date, 5) name and percentage of added materials** | **Statement of whether the Patent proprietary formula used in the trial is for a condition that is identical to the publicly available Reference (Y/N)** | **Chemical analysis reported? (Y/N)** | **Quality control reported? (Y/N)** |
| --- | --- | --- | --- | --- | --- | --- | --- | --- |
| Huai w 2009 | Ganmao qingre keli[感冒清热颗粒] | AURTIs | 3times/day, 6g/time | *Schizonepetae Herba(Jingjie), Menthae Haplocalycis Herba(Bohe), Saposhnikoviae Radix(Fangfeng), Bupleuri Radix(Chaihu), Perillae Folium(Zisuye), Puerariae Lobatae Radix(Gegen), Platycodonis Radix(Jiegeng), Armeniacae Semen Amarum(Xingren), Angelicae Dahuricae Radix(Baizhi), Corydalis Bungeanae Herba(Kudiding), Phragmitis Rhizoma(Lugen)*  **[[Pharmacopeia]**. Extract the volatile oil from the Jingjie, Bohe, Zisuye, and collect the aqueous solution after distillation in a separate vessel; decoct the dregs with the remaining eight flavors such as anti-feng with water for two times, combine the decoction, filter through, and combine the filtrate with the above aqueous solution. The combined solution is concentrated into clear paste with relative density of 1.32~1.35 (50℃); take the clear paste, add sucrose, dextrin and ethanol in appropriate amount, make granules, dry, add the above volatile oil, mix well, make 1600g [specification (1)]; or add auxiliary materials in appropriate amount, mix well, dry, add the above volatile oil, mix well, make 800g [specification (2)] or 533g [specification (3)] ( Without cane sugar); or the combined solution is concentrated under reduced pressure to a relative density of 1.08 ~ 1.10 (55 ℃), spray drying, made of dry paste powder, take the dry paste powder, add the appropriate amount of lactose, mix, add the above volatile oil, mix, made of particles 400g [specifications (4)], that is, (with lactose). | Added materials: Sucrose, dextrin and ethanol  Other information: NA | Y | N | N |
| Xue XF 2012 | Anti-viral Oral Liquid[抗病毒口服药] | AURTIs / Wind-heat cold | 3times/day, 10ml/time | *Isatidis Radix(Banlangen), Gypsum Fibrosum(Shigao), Phragmitis Rhizoma(Lugen), Rehmanniae Radix(Dihuang), Curcumae Radix(Yujin)25g, Anemarrhenae Rhizoma(Zhimu), Acori Tatarinowii Rhizoma(Shichangpu), Pogostemonis Herba(Guanghuoxiang), Forsythiae Fructus(Lianqiao)*  **[[Pharmacopeia]**Add water and decoct twice, the first time for 3 hours, collect the volatile oil, and cover with hydroxypropyl betacyclodextrin, or the first time for 1.5 hours (collect both volatile oil and volatile oil emulsion); the second time for 1 hour and 20 minutes, filter through, filtrate combined, concentrate to the appropriate amount, add more than 85% ethanol to make the alcohol content of 70%, let stand, filter through, filtrate recovered ethanol and concentrate to the appropriate amount, add volatile oil inclusion and Add volatile oil, volatile oil emulsion and appropriate amount of honey, sucrose, orange flavor, sodium cyclamate or add volatile oil, volatile oil emulsion and appropriate amount of honey, sucrose; adjust the pH value with 10% sodium hydroxide solution, filter through; add water to 1000ml, mix well, filter through, potting, sterilization, that is. | Added materials: Dextrin, honey, cane sugar, orange flavor, sodium cyclamate (or add volatile oil, volatile oil emulsion and appropriate amount of honey, cane sugar), 10% sodium hydroxide  Other information: NA | Y | N | N |
| Li HQ  2022 | Anti-viral Oral Liquid[抗病毒口服药] | Influenza, common cold | NR |  | Prepared by Guangzhou Xiangxue Pharmaceutical Co., Ltd  Added materials: Dextrin, honey, cane sugar, orange flavor, sodium cyclamate (or add volatile oil, volatile oil emulsion, and the appropriate amount of honey, cane sugar), 10% sodium hydroxide  Other information: NA | Y | N | N |
| Pan XS 2014 | Siji Anti-viral combination[四季抗病毒合剂] | AURTIs | 3times/day, 15mL/time | *Houttuyniae Herba(Yuxingcao), Platycodonis Radix(Jiegeng), Mori Folium(Sangye), Forsythiae Fructus(Lianqiao), Schizonepetae Herba(Jingjie), Menthae Haplocalycis Herba(Bohe), Perillae Folium(Zisuye), Armeniacae Semen Amarum(Xingren), Phragmitis Rhizoma(Lugen), Chrysanthemi Flos(Juhua), Glycyrrhizae Radix Et Rhizoma(Gancao)*  *NA* | Prepared by Shaanxi Haitian Pharmaceutical Co., Ltd., SFDA approval number: Z20027,669  Added materials: Sucrose, sodium benzoate  Other information: NA | Y | N | N |
| Wong 2012 | Yinqiao san[银翘散] | AURTIs | 2 times/day, 1bag/time, 7g/bag | *Forsythiae Fructus(Lianqiao), Lonicerae Japonicae Flos(Jinyinhua), Platycodonis Radix(Jiegeng), Menthae Haplocalycis Herba(Bohe), Glycyrrhizae Radix Et Rhizoma(Gancao), Schizonepetae Herba(Jingjie), Sojae Semen Praeparatum(Dandouchi), Arctii Fructus(Niubangzi), Phragmitis Rhizoma(Lugen), Lophatheri herba(Danzhuye)*  **[[Pharmacopeia]**Crushed into fine powder, sieved and mixed | No other substances added  Other information: NA | Y | N | Y |
| Sun JF 2014 | Qingre huashi liquid[清热化湿口服液] | AURTIs/damp-heat colds | 3times/day, 20ml/time | *Scutellariae Radix(Huangqin), Pinelliae Rhizoma Praeparatum(Fabanxia), Talci Pulvis(Huashifen), Artemisiae Annuae Herba(Qinghao), Sojae Semen Praeparatum(Dandouchi), Belamcandae Rhizoma(Shegan), Phragmitis Rhizoma(Lugen), Benincasa hispida(Dongguazi), Coicis Semen(Yiyiren), Armeniacae Semen Amarum(Xingren), Descurainiae Semen Lepidii Semen(Tinglizi), Eriobotryae Folium(Pipaye), Curcumae Radix(Yujin)*  *NA* | Sichuan Provincial Hospital of Traditional Chinese Medicine In-hospital preparation  Other information: NA | N | N | N |
| Zhou JC 2013 | Qingjiekanggankeli[清解抗感颗粒] | AURTIs/Superficial cold and internal heat | 3times/day, 2bags/time, 4g/bag, | Wang Yongyan academician's clinical experience prescription, *Schizonepetae Herba(Jingjie), Scutellariae Radix(Huangqin), Saposhnikoviae Radix(Fangfeng), Menthae Haplocalycis Herba(Bohe), Notopterygii Rhizoma Et Radix(Qianghuo), Gardeniae Fructus(Zhizi), Peucedani Radix(Qianhu), Platycodonis Radix(Jiegeng), Armeniacae Semen Amarum(Xingren), Phragmitis Rhizoma(Lugen)*.  *NA* | Wuhu Greenleaf Pharmaceutical Co., Ltd, Batch No.:20090935  Other information: NA | N | N | N |

AURTIs: Acute upper respiratory tract infection

**Supplementary Table 4 Details of fixed Chinese herbal medicine formula included in the trials**

| **ID** | **Diagnosis/**  **syndrome differentiation** | **Name, source, processing method, and dosage of each medical substance** | **The authentication method of each ingredient and each ingredient and how, when, where, and by whom it was conducted; statement of whether any voucher specimen was retained, and if so, where they were kept and whether they are accessible** | **Principles, rationale, and interpretation of forming the formula** | **Production methods and how to take the medication** | **Safety assessment of formulations** | **References as to the efficacy of the formula** | **Chemical analysis reported?(Y/N)** | **Quality control reported?(Y/N)** |
| --- | --- | --- | --- | --- | --- | --- | --- | --- | --- |
| Zhang XT 2016 | Wind-heat cold | *Lonicerae Japonicae Flos(Jinyinhua)15g, Forsythiae Fructus(Lianqiao)15g, Arctii Fructus(Niubangzi)10g, Menthae Haplocalycis Herba(Bohe)10g, Schizonepetae Herba(Jingjie)5g, Sojae Semen Praeparatum(Dandouchi)10g, Platycodonis Radix(Jiegeng)10g, Lophatheri herba(Danzhuye)5g, Phragmitis Rhizoma(Lugen)20g, Glycyrrhizae Radix Et Rhizoma(Gancao)10g.* | NA | Y, *P. communis* as the assistant medicinal | Decoction with water, 1 dose/day, 2 times/day, double the dose for severe hyperthermia | NA | NA | N | N |
| Xu XF 2013 | AURTIs, gas partition | *Gypsum Fibrosum(Shigao)50 g, Menthae Haplocalycis Herba(Bohe)10 g, Phragmites Rhizoma(Lugen)3 g, Divina Comoedia Fricta(Shenqu)10 g* | NA | Y, *P. communis* as the  minister medicinal | Decoction with water, drink as tear, 1 dose/day | NA | NA | N | N |
| Sun JF 2014 | AURTIs/  Wind-heat cold | *Mori Folium(Sangye)10g, Chrysanthemi Indici Flos(Yejuhua)10g, Lonicerae Japonicae Flos(Jinyinhua)10g, Forsythiae Fructus(Lianqiao)10g, Menthae Haplocalycis Herba(Bohe)10g, Schizonepetae Herba(Jingjie)10g, Sojae Semen Praeparatum(Douchi)10g, Arctii Fructus(Niubangzi)10g, Platycodonis Radix(Jiegeng)10g, Lophatheri Herba(Danzhuye)10g, Phragmites Rhizoma(Lugen)10g, Glycyrrhizae Radix Et Rhizoma(Gancao)3g.* | NA | Y, *P. communis* as the assistant medicinal | Chinese herbal medicine granules, 1 dose/day, 4 times/day | Pharmacological tests | NA | N | N |
| Song DS 2021 | AECOPD | *Pinelliae Rhizoma Praeparatum(Fabanxia)12g, Atractylodis Macrocephalae Rhizoma(Baizhu)10g, Persicae Semen(Taoren)10g, Glycyrrhizae Radix Et Rhizoma Praeparata Cum Melle(Zhigancao)6g, Phragmitis Rhizoma(Lugen)30g, Ephedrae Herba(Mahuang)10g, Coicis Semen(Yiyiren)20g, Hiems Cucumis Kernel(Dongguaren)15g, Platycodonis Radix(Jiegeng)12g, Bolbostemmatis Rhizoma(Tubeimu)12g, Poria(Fuling)15g, Armeniacae Semen Amarum(Xingren)6g, Citri Reticulatae Pericarpium(Chenpi)10g, Aurantii Fructus(Zhiqiao)10g.* | NA | Y | Decoct 2 times with water to 300 mL of the drug mixture. 1 dose/day, 2 times/day | Pharmacology Research | NA | N | N |
| Sun L 2022 | Fever | *Gypsum Fibrosum(Shigao), Anemarrhenae Rhizoma(Zhimu), Bupleuri Radix(Chaihu), Puerariae Lobatae Radix(Gegen), Scutellariae Radix(Huangqin), Cimicifugae Rhizoma(Shengma), Menthae Haplocalycis Herba(Bohe), Phragmitis Rhizoma(Lugen), Glycyrrhizae Radix Et Rhizoma(Gancao).* | NA | Y, *P. communis* as the assistant medicinal | Prepared by the pharmacy of Wuhu Traditional Chinese Medicine Hospital. 1 bag/day, 3 times/day | NA | NA | N | N |
| Li YP 2022 | Influenza | *Bupleuri Radix(Chaihu)15g, Scutellariae Radix(Huangqin)15g, Gypsum Fibrosum(Shigao)50g, Glycyrrhizae Radix Et Rhizoma Praeparata Cum Melle(Zhigancao)10g, Ephedrae Herba(Mahuang)5g, Lonicerae Japonicae Flos(Jinyinhua)25g, Forsythiae Fructus(Lianqiao)15g, Phragmitis Rhizoma(Lugen)15g, Armeniacae Semen Amarum(Xingren)15g, Menthae Haplocalycis Herba(Bohe)10g, Schizonepetae Herba(Jingjie)15g, Arctii Fructus(Niubangzi)15g, Zingiberis Rhizoma Recens(Shengjiang)15g, Jujubae Fructus(Dazao)15g.* | NA | Y | Prepared by the Smart Pharmacy of the Second Hospital of Liaoning University of Traditional Chinese Medicine. 1 dose/day, 2 times/day | NA | NA | N | N |
| Li J 2020 | AECOPD/  external cold and internal drinking | The Jinkui Yaolue. *Belamcandae Rhizoma(Shegan)20g, Ephedrae Herba(Mahuang)15g, Ginkgo Semen(Baiguo)20g, Phragmitis Rhizoma(Lugen)25g, Chebulae Fructus(Hezi)20g, Perillae Fructus(Zisuzi)15g, Armeniacae Semen Amarum(Xingren)20g, Glycyrrhizae Radix Et Rhizoma(Gancao)15g.* | NA | Y, *P. communis* as the assistant medicinal | Prepared by decoction room of Nangang Branch of Heilongjiang Provincial Institute of Traditional Chinese Medicine. 2 times/day, 1 time/150ml, 30 minutes after meals in the morning and evening. | Pharmacology Research | NA | N | N |
| Hu QY 2019 | Acute sinusitis with heat in the lung meridian | Thousand-Golden-Prescriptions. *Phragmitis Rhizoma(Lugen)15g, Persicae Semen(Taoren)10g, Coicis Semen(Yiyiren)20g, Hiems Cucumis Kernel(Dongguaren)12g, Scutellariae Radix(Huangqin)8 g, Patrinia(Baijiangcao)30g, Acori Tatarinowii Rhizoma(Shichangpu)8g, Angelicae Dahuricae Radix(Baizhi)10g, Gleditsiae Spina(Zaojiaoci)10g, Liquidambaris Fructus(Lulutong)10g, Magnoliae Flos(Xinyi)10 g, Centipeda Herba(Ebushicao)10g, Platycodonis Radix(Jiegeng)10g, Glycyrrhizae Radix Et Rhizoma(Gancao)6g.* | NA | Y, *P. communis* as the sovereign medicinal | The drug was first soaked in cold water for 20 min and then decocted 2 times to obtain a total of about 400 ml of juice. 1 dose/day, 2 times/day, taken in the morning and evening. | NA | NA | N | N |
| Yang LY 2019 | Fever | *Scutellariae Radix(Huangqin)30g, Platycodonis Radix(Jiegeng)3g, Phragmitis Rhizoma(Lugen)12g, Imperatae Rhizoma(Baimaogen)12g, Farfarae Flos(Kuandonghua)9g, Atractylodis Rhizoma(Cangzhu)20g, Armeniacae Semen Amarum(Xingren)9g.* | NA | Y | Prepared by Shenzhen Sanjiu Pharmaceutical Company | NA | NA | N | N |
| Xue JH 2017 | Fever | *Armeniacae Semen Amarum(Xingren)9g, Farfarae Flos(Kuandonghua)9g, Imperatae Rhizoma(Baimaogen)12g, Phragmitis Rhizoma(Lugen)12g, Platycodonis Radix(Jiegeng)3g, Scutellariae Radix(Huangqin)30g, Atractylodis Rhizoma(Cangzhu)20g.* | NA | Y | Provided by China Resources Sanjiu Pharmaceutical Co. Chinese Medicine No Decoction Granules, 3 times/day, 1 bag/time | NA | NA | N | N |
| Cheng Y 2016 | AURTIs, | Professor Chen Jianjie's Experienced Formula. *Professor Chen Jianjie's Experienced Formula. Scutellariae Radix(Huangqin)30g, Platycodonis Radix(Jiegeng)3g, Phragmitis Rhizoma(Lugen)12g, Imperatae Rhizoma(Baimaogen)12g, Farfarae Flos(Kuandonghua)9g, Atractylodis Rhizoma(Cangzhu)20g, Armeniacae Semen Amarum(Xingren)9g.* | NA | Y | Provided by China Resources Sanjiu Pharmaceutical Co. | NA | NA | N | N |
| Zhang ZX 2017 | AECOPD with phlegm-heat congestion in the lung and deficiency of both qi and yin | *Houttuyniae Herba(Yuxingcao)30g, Pinelliae Rhizoma Praeparatum(Fabanxia)10g, Bambusae Caulis In Taenias(Zhuru)10g, Fritillariae Thunbergii Bulbus(Zhebeimu)15g, Mori Cortex(Sangbaipi)15g, Citri Reticulatae Pericarpium(Chenpi)10g, Poria(Fuling)15g, Aurantii Fructus Immaturus(Zhishi)10g, Adenophorae Radix(Nansahshen)30g, Ophiopogonis Radix(Maidong)15g, Pheretima(Dilong)15g, Salviae Miltiorrhizae Radix Et Rhizoma(Danshen)15g, Phragmitis Rhizoma(Lugen)15g, Amomi Fructus(Sharen)10g, Platycodonis Radix(Jiegeng)10g, Glycyrrhizae Radix Et Rhizoma(Gancao)10g.* | NA | Y | The first decoction is made by adding 400m1 of water and letting it soak for 30 minutes. Then boil the mixture for 20 minutes and extract? 150ml of juice; the second decoction is made by adding 300m1 of water, boiling for 20 minutes, and taking 150m1 of juice, mixing the two decoctions. 1 dose/day, 3 times/day, 1 bag/time, 1 bag/100ml, take after meals. | NA | NA | N | N |
| Wei ZJ 2014 | AECOPD | Professor Wan qinan's Experienced Formula. *Houttuyniae Herba(Yuxingcao)30g, Phragmitis Rhizoma(Lugen)20g, Rehmanniae Radix(Dihuang)10g, Citri Reticulatae Pericarpium(Chenpi)10g, Taraxaci Herba(Pugongying)30g, Pseudostellariae Radix(Taizishen)20g, Salviae Miltiorrhizae Radix Et Rhizoma(Danshen)10g, Glycyrrhizae Radix Et Rhizoma(Gancao)6g, Mori Cortex(Sangbaipi)15g, Ophiopogonis Radix(Maidong)15g, Trichosanthis Fructus(Gualou)15g, Nelumbinis Folium(Heye)10g, Platycodonis Radix(Jiegeng)10g, Bupleuri Radix(Chaihu)10g.* | NA | Y, *P. communis* as the minister medicinal | Same as above | Pharmacology Research | NA | N | N |
| Zhang ZX 2016 | AECOPD with phlegm-heat congestion in the lung and deficiency of both qi and yin | *Houttuyniae Herba(Yuxingcao)30g, Asteris Radix Et Rhizoma(Ziwan)15g, Farfarae Flos(Kuandonghua)15g, Trichosanthis Fructus(Gualou)15g, Mori Cortex(Sangbaipi)15g, Glehniae Radix(Beishashen)30g, Ophiopogonis Radix(Maidong)15g, Schisandrae Chinensis Fructus(Wuweizi)10g, Citri Reticulatae Pericarpium(Chenpi)10g, Poria(Fuling)15g, Phragmitis Rhizoma(Lugen)30g, Anemarrhenae Rhizoma(Zhimu)10g, Salviae Miltiorrhizae Radix Et Rhizoma(Danshen)15g, Paeoniae Radix Rubra(Chishao)15g, Platycodonis Radix(Jiegeng)10g, Glycyrrhizae Radix Et Rhizoma(Gancao)10g.* | NA | Y | 1 dose/day, 3 times/day, 1 bag/time, take after meals | NA | NA | N | N |
| Sun YT 2014 | Influenza (wind-heat attacking the defense system, wind-cold binding the exterior, heat-toxin invading the lungs) | *Gypsum Fibrosum(Shigao)15g, Anemarrhenae Rhizoma(Zhimu)10g, Isatidis Radix(Banlangen)15g, Forsythiae Fructus(Lianqiao)10g, Phragmitis Rhizoma(Lugen)10g, Rehmanniae Radix(Dihuang)10g, Curcumae Radix(Yujin)10g, Acori Tatarinowii Rhizoma(Shicangpu)10g, Pogostemonis Herba(Guanghuoxiang)10g.* | NA | Y | Provided by Sichuan New Green Pharmaceutical Technology Development Co. Chinese medicine no decoction granules, 2 times/day | NA | NA | N | N |
| Wang FW 2007 | Influenza | *Lonicerae Japonicae Flos(Jinyinhua)15g, Forsythiae Fructus(Lianqiao)15g, Platycodonis Radix(Jiegeng)10g, Phragmitis Rhizoma(Lugen)10g, Puerariae Lobatae Radix(Gegen)10g, Bupleuri Radix(Chaihu)10g, Scutellariae Radix(Huangqin)20g, Scrophulariae Radix(Xuanshen)15g, Belamcandae Rhizoma(Shegan)10 g, Isatidis Radix(Banlangen)15g, Isatidis Folium(Daqingye)15g, Mori Folium(Sangye)10g, Trichosanthis Fructus(Gualou)10g, Glycyrrhizae Radix Et Rhizoma(Gancao)10g, Armeniacae Semen Amarum(Xingren)10g, Galli Gigerii Endothelium Corneum(Jineijin)15g, Cinnamomi Ramulus(Guizhi)4g.* | NA | Y | Decoction with water, 1 dose/day, 2 times/day | NA | NA | N | N |
| Li HY 2022 | AECOPD sputum-dampness blocking the lung with qi and yin deficiency | *Ginseng Radix Et Rhizoma(Renshen)10g, Glehniae Radix(Beishashen)15g, Perillae Folium(Zisuye)6g, Armeniacae Semen Amarum(Xingren)10g, Platycodonis Radix(Jiegeng)6g, Arctii Fructus(Niubangzi)10g, Magnoliae Officinalis Cortex(Houpo)6g, Phragmitis Rhizoma(Lugen)15g, Coicis Semen(Yiyiren)15g, Pinelliae Rhizoma Praeparatum(Fabanxia)15g, Plantaginis Semen(Cheqianzi)15g, Poria(fuling)15g, Glycyrrhizae Radix Et Rhizoma Praeparata Cum Melle(Zhigancao)6g, Myristicae Semen(Roudoukou)4g, Zingiberis Rhizoma Recens(Shengjiang)10g.* | NA | Y | Decoction of 400 ml, 1 dose/day, 2 times/day, 200 ml/time | NA | NA | N | N |
| Zheng YL 2019 | AECOPD Phlegm-heat congestion | *Ephedrae Herba(Mahuang)9g, Gypsum Fibrosum(Shengshigao)30g, Trichosanthis Fructus(Gualou)30g, Phragmitis Rhizoma(Lugen)30g, Salviae Miltiorrhizae Radix Et Rhizoma(Danshen)30 g, Armeniacae Semen Amarum(Xingren)10g, Magnoliae Officinalis Cortex(Houpo)10g, Scutellariae Radix(Huangqin)10g, Pinelliae Rhizoma(Banxia)10g, Pheretima(Dilong)10g, Glycyrrhizae Radix Et Rhizoma(Gancao)10g, Citri Exocarpium Rubrum(Juhong)6g.* | NA | Y | Provided by the preparation room. 150 ml/bag, 2 times/day, 150 ml/time | NA | NA | N | N |
| Wang DY 2020 | AECOPD  Phlegm-heat obstructing the lungs | *Mori Cortex(Sangbaipi)10g, Scutellariae Radix(Huangqin)10g, Fagopyri Dibotryis Rhizoma(Jinqiaomai)15g, Bolbostemmatis Rhizoma(Tubeimu)10g, Pinelliae Rhizoma Praeparatum(Fabanxia)6g, Anemarrhenae Rhizoma(Zhimu)10g, Armeniacae Semen Amarum(Xingren)10g, Platycodonis Radix(Jiegeng)6g, Houttuyniae Herba(Yuxingcao)15g, Phragmitis Rhizoma(Lugen)15g, Salviae Miltiorrhizae Radix Et Rhizoma(Danshen)15g, Glycyrrhizae Radix Et Rhizoma(Gancao)6g, Trichosanthis Fructus(Gualou)10g.* | NA | Y | Decocted with water, 1 time/day | Pharmacology Research | NA | N | N |
| Chen H 2014 | AECOPD phlegm-heat blocking lung | *Mori Cortex(Sangbaipi)20g, Fritillariae Thunbergii Bulbus(Zhebeimu)30g, Pinelliae Rhizoma Praeparatum(Fabanxia)10g, Coptidis Rhizoma(Huanglian)6g, Trichosanthis Fructus(Gualou)20g, Phragmitis Rhizoma(Lugen)30g, Platycodonis Radix(Jiegeng)10g, Persicae Semen(Taoren)10g, Citri Reticulatae Pericarpium(Chenpi)10g, Poria(Fuling)10g, Atractylodis Macrocephalae Rhizoma(Baizhu)10g, Zingiberis Rhizoma Recens(Shengjiang)6g, Jujubae Fructus(Dazao)10g, Glycyrrhizae Radix Et Rhizoma Praeparata Cum Melle(Zhigancao)6g.* | NA | Y | Take about 200ml of water, 2 times orally  Formula developed by a department | NA | NA | N | N |
| Shi JP 2010 | AECOPD phlegm-heat stasis in lung and lung spleen qi deficiency evidence | Six Jun Zi Tang (Women's Good Recipe) combined with Reed Stem Tang (Thousand-Golden-Prescriptions). *Codonopsis Radix(Dangshen)12g, Poria(Fuling)10g, Atractylodis Macrocephalae Rhizoma(Baizhu)10g, Citri Reticulatae Pericarpium(Chenpi)10g, Pinelliae Rhizoma Praeparatum(Fabanxia)10g, Phragmitis Rhizoma(Lugen)20g, Hiems Cucumis Kernel(Dongguaren)20g, Coicis Semen(Yiyiren)20g, Persicae Semen(Taoren) 10g, Chuanxiong Rhizoma(Chuanxiong)10g.* | NA | Y | Decocted with water, 1 dose/day, 2 times/day | NA | NA | N | N |
| Big Dipper 2019 | Acute bronchitis with phlegm-heat congestion in the lung | *Gypsum Fibrosum(Shigao)25g, Mori Folium(Sangye)25g, Armeniacae Semen Amarum(Xingren)20g, Chrysanthemi Flos(Juhua)20g, Platycodonis Radix(Jiegeng)15g, Fritillariae Thunbergii Bulbus(Zhebeimu)15g, Forsythiae Fructus(Lianqiao)15g, Lonicerae Japonicae Flos(Jinyinhua)15g, Trichosanthis Fructus(Gualou)15g, Phragmitis Rhizoma(Lugen)15g, Saposhnikoviae Radix(Fangfeng)10g, Citri Reticulatae Pericarpium(Chenpi)10g, Ephedrae Herba(Mahuang)10g, Mori Cortex(Sangbaipi)10g, Lycii Cortex(Digupi)10g, Glycyrrhizae Radix Et Rhizoma Praeparata Cum Melle(Zhigancao)8g.* | NA | Y | Add 300mL of water and decoct to 150mL, take in the morning and evening. | Pharmacology Research | NA | N | N |
| Zong Y 2020 | Influenza | *Lonicerae Japonicae Flos(Jinyinhua)12g, Forsythiae Fructus(Lianqiao)12g, Mori Folium(Sangye)12g, Chrysanthemi Flos(Juhua)12g, Phragmitis Rhizoma(Lugen)12g, Isatidis Radix(Banlangen)12g, Sojae Semen Praeparatum(Dandouchi)9g, Divina Comoedia Fricta(Shenqu)12g, Arctii Fructus(Niubangzi)12g, Tetrastigma hemsleyanum Diels et Gilg(Sanyeqing)10g, Menthae Haplocalycis Herba(Bohe)5g, Puerariae Lobatae Radix(Gegen)9 g, Glycyrrhizae Radix Et Rhizoma(Gancao)5g.* | NA | Y | Decoct the medicine twice with water for 15 min each, take the juice and mix, 1 dose/day, 2 times/day, and take in the morning and evening. | NA | NA | N | N |
| Chang QJ 2016 | AECOPD | *Wenbing Tiaobian. Gypsum Fibrosum(Shigao)30g, Trichosanthis Fructus(Gualou)15g, Rhei Radix Et Rhizoma(Dahaung)10g , Anemarrhenae Rhizoma(Zhimu)15g, Armeniacae Semen Amarum(Xingren)10g, Scutellariae Radix(Huangqin)15g, Houttuyniae Herba(Yuxingcao)15g, Fritillariae Thunbergii Bulbus(Zhebeimu)15g, Descurainiae Semen Lepidii Semen(Tinglizi)15g, Phragmitis Rhizoma(Lugen)25g, Pheretima(Dilong)10g, Ophiopogonis Radix(Maidong)15g, Adenophora stricta Miq.(Shashen)15g, Glycyrrhizae Radix Et Rhizoma(Gancao)15g.* | NA | Y | Decoction of 300 ml of juice, 1 dose/day, 2 times/day, taken in the morning and evening. | Pharmacology Research | NA | N | N |
| Xu GJ 2014 | AURTIs | *Lonicerae Japonicae Flos(Jinyinhua)10g, Schizonepetae Herba(Jingjie)10g, Inversa Grass(Daokoucao)5g, Salix gracilior (Siuz.) Nakai(Xisiliu)5g, Phragmitis Rhizoma(Lugen)10g, Saposhnikoviae Radix(Fangfeng)6g, Forsythiae Fructus(Lianqiao)6g.* | NA | N | Add 1000 mL of water and decoct to obtain 300 mL of juice, 2 times/day, take in the morning and evening. | Pharmacology Research | NA | N | N |

NA: Not available

1. *communis*: Phragmitis Rhizoma

*sovereign, minister, assistant, and courier [君臣佐使]mean the ingredients in a formula or prescription that have different roles. Sovereign medicinal[君药] is the ingredient that provides the principal curative action on the main pattern/syndrome or primary symptom. Minister medicinal[臣药] is the ingredient that helps strengthen the principal. Assistant medicinal[佐药] is the ingredient that treats the combined pattern/syndrome, relieves secondary symptoms, or tempers the action of the sovereign ingredient when the latter is too potent. Courier medicinal[使药] is the ingredient that directs action to the affected meridian/channel or site.

AURTIs: Acute upper respiratory tract infections

AECOPD: Acute exacerbation of chronic obstructive pulmonary disease

**Supplementary Table 5 Details of individual Chinese medicines formula included in the trials.**

| **ID** | **Diagnosis** | Name, source, processing method, and dosage of each medical substance | The authentication method of each ingredient and each ingredient and how, when, where,and by whom it was conducted; statement of whether any voucher specimen was retained,and if so, where they were kept and whether they are accessible | Principles, rationale, and interpretation of forming the formula* | **Production method and how to take the medication** | Safety assessment of formulations | References as to the efficacy of the formula | Chemical analysis reported? (Y/N) | Quality control reported? (Y/N) | **How, when and by whom herbs are added or reduced？** |
| --- | --- | --- | --- | --- | --- | --- | --- | --- | --- | --- |
| Wang QM 2010 | AURTIs | *Lonicerae Japonicae Flos(Jinyinhua)10g, Forsythiae Fructus(Lianqiao)10g, Platycodonis Radix(Jiegeng)10g, Arctii Fructus(Niubangzi)6g, Schizonepetae Herba(Jingjie)6g, Phragmitis Rhizoma(Lugen)10g, Taraxaci Herba(Pugongying)10g, Menthae Haplocalycis Herba(Bohe)10g, Lophatheri herba(Danzhuye)6g.* | NA | Y, *P. communis* as the Assistant medicinal | Decoction with water, 1 dose/day, 2 times/day | NA | NA | N | N | Add or subtract Chinese herbs according to symptoms, other information not available |
| Guo, XH 2009 | Bacterial tonsillitis | *Lonicerae Japonicae Flos(Jinyinhua)10g, Forsythiae Fructus(Lianqiao)12g, Platycodonis Radix(Jiegeng)9g, Menthae Haplocalycis Herba(Bohe)9g,Lophatheri herba(Danzhuye)6g, Arctii Fructus(Niubangzi)9g, Schizonepetae Herba(Jingjie)6g,Sojae Semen Praeparatum(Dandouchi)6g,Isatidis Radix(Banlangen)9g,Phragmitis Rhizoma(Lugen)12g,Imperatae Rhizoma(Baimaogen)15-20g,Radix Et Rhizoma(Gancao)6g.* | NA | Y | Decoction with water, 1-2 doses/day, soak the drug in cold water for 20 min, after opening before the martial fire, add mint for 2 min before the civil fire for 7 min, take out the liquid, divide into two doses, 12 h once for mild cases, 6 h once for severe cases. | NA | NA | N | N | Same as above |
| Zhang MX 2006 | AURTIs | *Gypsum Fibrosum(Shigao) 20-30g, Anemarrhenae Rhizoma(Zhimu)15g, Lonicerae Japonicae Flos(Jinyinhua)20g, Forsythiae Fructus(Lianqiao)15g, Scutellariae Radix(Huangqin)10g, Bupleuri Radix(Chaihu)10g, Puerariae Lobatae Radix(Gegen)15g, Phragmitis Rhizoma(Lugen)20g, Rehmanniae Radix(Dihuang)15g.* | NA | Y | NA | NA | NA | N | N | Same as above |
| Zhou ZW 2021 | Acute pharyngitis | *Rehmanniae Radix(Dihuang)20g, Scrophulariae Radix(Xuanshen)10g, Ophiopogonis Radix(Maidong)15g, Scutellariae Radix(Huangqin)10g, Platycodonis Radix(Jiegeng)10g, Phragmitis Rhizoma(Lugen)20g, Achyranthis Bidentatae Radix(Niuxi)15g, Uncariae Ramulus Cum Uncis(Gouteng)15g, Menthae Haplocalycis Herba(Bohe)5g, Radix Et Rhizoma(Gancao)5g.* | NA | Y | Decoction with water, 1 dose/day, 2 times/day | NA | NA | N | N | Same as above |
| Luo HL2019 | Acute cough | *Mori Cortex(Sangbaipi)20g, Phragmitis Rhizoma(Lugen)30g, Houttuyniae Herba(Yuxingcao)30g, Trichosanthis Radix(Tianhuafen)30g, Trichosanthis Fructus(Gualou)5g, Forsythiae Fructus(Lianqiao)5g, Fritillariae Thunbergii Bulbus(Zhebeimu)5g, Raphani Semen(Laifuzi)5g, Arctii Fructus(Niubangzi)15g, Platycodonis Radix(Jiegeng)10g, Menthae Haplocalycis Herba(Bohe)5g, Radix Et Rhizoma(Gancao)5g.* | NA | Y | Decoct in water for about half an hour, 250 mL, and re-decoct. Take in the morning and evening. | NA | NA | N | N | Same as above |
| Li CY 2022 | AECOPD | *Ephedrae Herba(Mahuang)10g, Fritillariae Thunbergii Bulbus(Zhebeimu)15g, Phragmitis Rhizoma(Lugen)15g, Fagopyri Dibotryis Rhizoma(Jinqiaomai)10g, Mori Cortex(Sangbaipi)10g, Platycodonis Radix(Jiegeng)10g, Pinelliae Rhizoma Praeparatum(Fabanxia)10g, Armeniacae Semen Amarum(Xingren)6g, Scutellariae Radix(Huangqin)10g, Descurainiae Semen Lepidii Semen(Tinglizi)10g, Radix Et Rhizoma(Gancao)3g.* | NA | Y | Provided by the Chinese medicine department of Hainan Provincial Hospital, 1 dose/day | NA | NA | N | N | Same as above |
| Hui P 2012 | Influenza A (H1N1) | *Lonicerae Japonicae Flos(Jinyinhua)12g, Forsythiae Fructus(Lianqiao)12g, Menthae Haplocalycis Herba(Bohe)10g, Arctii Fructus(Niubangzi)10g, Belamcandae Rhizoma(Shegan)10g, Isatidis Folium(Daqingye)30g, Isatidis Radix(Banlangen)20g, Platycodonis Radix(Jiegeng)10g, Phragmitis Rhizoma(Lugen)10g, Radix Et Rhizoma(Gancao)6g.* | NA | Y | Decoction to 150-200mL per dose, 2 doses/day, 1 dose in the morning and 1 dose in the evening | NA | NA | N | N | Same as above |
| Hong LL 2020 | AECOPD | *Polygoni Cuspidati Rhizoma Et Radix(Huzhang)20g, Phragmitis Rhizoma(Lugen)20g, Coicis Semen(Yiyiren)20g, Teucrium Viscidum Bl. Bijdr(Feixingcao)10g, Perillae Fructus(Zisuzi)10g, Farfarae Flos(Kuandonghua)10g, Asteris Radix Et Rhizoma(Ziwan)10g, Poria(Fuling)15g, Salviae Miltiorrhizae Radix Et Rhizoma(Danshen)15g, Spatholobi Caulis(Jixueteng)15g, Chuanxiong Rhizoma(Chuanxiong)9g, Paeoniae Radix Rubra(Chishao)15g, Pheretima(Dilong)10g, Platycodonis Radix(Jiegeng)6g, Houttuyniae Herba(Yuxingcao)15g, Pinelliae Rhizoma Praeparatum Cum Zingibere Et Alumine(Jiangbanxia)10g, Descurainiae Semen Lepidii Semen(Tinglizi)10g, Rubiae Radix Et Rhizoma(Qiancao)10g, Arnebiae Radix(Zicao)10g, Moutan Cortex(Mudanpi)10g, Citri Reticulatae Pericarpium(Chenpi)9g, Radix Et Rhizoma(Gancao)6g.* | NA | Y | Provided by the pharmacy of Nantong Hospital of Traditional Chinese Medicine, Jiangsu Province. 1 dose/day | NA | NA | N | N | Same as above |
| Ye D 2020 | Acute bronchitis | *Phragmitis Rhizoma(Lugen)20g, Trichosanthis Fructus(Gualou)15g, Mori Cortex(Sangbaipi)15g, Notopterygii Rhizoma Et Radix(Qianghuo)15g, Platycodonis Radix(Jiegeng)15g, Armeniacae Semen Amarum(Xingren)15g, Fritillariae Ussuriensis Bulbus(Pingbeimu)10g, Radix Et Rhizoma(Gancao)5g.* | NA | Y | The herbs were boiled to 400 mL before adding 1,000 ml of water, 2 times/day, 200 ml/time, with an interval of 30 min or more between doses of western medicine | NA | NA | N | N | Same as above |
| Chen XM 2016 | AURTIs | *Mori Folium(Sangye)15g, Chrysanthemi Flos(Juhua)15g, Forsythiae Fructus(Lianqiao)15g, Armeniacae Semen Amarum(Xingren)10g, Radix Et Rhizoma(Gancao)6g, Phragmitis Rhizoma(Lugen)30g, Platycodonis Radix(Jiegeng)15g.* | NA | Y | Decoction with water, 1 dose/day, 2 times/day, 200m l/time. | NA | NA | N | N | Same as above |
| Guo XX 2017 | Acute pharyngitis | A Brief Treatise on Epidemic Throat by Xia Chunnong, a Qing Dynasty thermitologist.  *Sojae Semen Praeparatum(Dandouchi)15g, Gardeniae Fructus(Zhizi)12g, Bubali Cornu(Shuiniujiao)15g, Platycodonis Radix(Jiegeng)15g, Arctii Fructus(Niubangzi)15g, Lasiosphaera Calvatia(Mabo)10g, Bombyx Batryticatus(Baijiangcan)10g, Cicadae Periostracum(Chantui)10g, Forsythiae Fructus(Lianqiao)10g, Lonicerae Japonicae Flos(Jinyinhua)10g, Lophatheri herba(Danzhuye)12g, Menthae Haplocalycis Herba(Bohe)6g, Phragmitis Rhizoma(Lugen)12g, Junci Medulla(Dengxingcao)10g, Radix Et Rhizoma(Gancao)6g.* | NA | Y | Decoction with water to 200 ml, 1 dose/day, 2 times/day | NA | NA | N | N | Same as above |

AURTIs: Acute upper respiratory tract infections

NA: Not available

*P. communis* : Phragmitis Rhizoma

sovereign, minister, assistant, and courier [君臣佐使]mean the ingredients in a formula or prescription that have different roles. Sovereign medicinal[君药] is the ingredient that provides the principal curative action on the main pattern/syndrome or primary symptom. Minister medicinal[臣药] is the ingredient that helps strengthen the principal. Assistant medicinal[佐药] is the ingredient that treats the combined pattern/syndrome, relieves secondary symptoms, or tempers the action of the sovereign ingredient when the latter is too potent. Courier medicinal[使药] is the ingredient that directs action to the affected meridian/channel or site.

AECOPD: Acute exacerbation of chronic obstructive pulmonary disease

**Supplementary Table 6. *Phragmites communis* versus *Placebo***

| **Outcome or subgroup title** | **No. of studies** | **No. of participants** | **Statistical method(random model)** | **Effect size** | **Quality of**  **Evidence(GRADE)** |
| --- | --- | --- | --- | --- | --- |
| **Symptoms** | | | | | |
| Cure rate | 4 | 436 | Risk Ratio(M-H, Random, 95% CI) *I^2^*=0 | 1.60[1.13,2.26] | low certainty |
| Antipyretic time | 2 | 181 | Mean Difference  (IV, 95% CI) *I^2^*=0% | -2.73[-4.85,-0.61] | low certainty |

**Supplementary Table 7*. Phragmites communis Plus Usual care* versus *Usual care***

| **Outcome or subgroup title** | **No. of studies** | **No. of participants** | **Statistical method**  **(random model)** | **Effect size** | **Quality of**  **Evidence(GRADE)** |
| --- | --- | --- | --- | --- | --- |
| **Symptoms** | | | | |  |
| Cure rate | 18 | 1426 | Risk Ratio (M-H, 95% CI) I^2^=0 | 1.55[1.35, 1.78] | low certainty |
| Combination therapy | 16 | 1248 | Risk Ratio (M-H, 95% CI) I^2^=0 | 1.58[1.35, 1.84] | low certainty |
| Symptomatic drugs | 1 | 58 | Risk Ratio (M-H, 95% CI) | 1.87[0.18, 19.47] | Very low certainty |
| Antiviral drugs | 1 | 120 | Risk Ratio (M-H, 95% CI) | 1.38[0.97, 1.98] | Very low certainty |
| TCM Syndrome scores | 8 | 535 | Std.Mean Difference  (IV, 95% CI) I^2^=90% | -1.34[-1.95, -0.74] | Very low certainty |
| Antipyretic time | 4 | 328 | Mean Difference  (IV, 95% CI) I^2^=97% | -19.31[-33.35, -5.27] | low certainty |
| **Pulmonary function-related outcomes** | | | | |  |
| FEV1 | 6 | 476 | Mean Difference  (IV, 95% CI) I^2^=0 | 0.19[0.13, 0.26] | low certainty |
| FEV1% | 7 | 434 | Mean Difference  (IV, 95% CI) I^2^=35% | 4.85[2.85, 6.86] | Very low certainty |
| FVC | 4 | 276 | Mean Difference (IV, 95% CI) I^2^=0 | 0.16[0.03, 0.28] | Very low certainty |
| FEV1/FVC | 7 | 516 | Std.Mean Difference  (IV, 95% CI) I^2^=90% | 0.82[0.24, 1.40] | Very low certainty |
| **Inflammatory factors outcomes** | | | | | |
| TNF-α | 2 | 180 | Mean Difference (IV, 95% CI) I^2^=13% | -0.31[-0.37, -0.26] | Very low certainty |
| IL-8 | 2 | 180 | Mean Difference (IV, 95% CI) I^2^=97% | -0.24[-0.41,-0.08] | Very low certainty |
| CRP | 4 | 290 | Mean Difference (IV, 95% CI) I^2^=99% | -10.52[-21.88, 0.84] | Very low certainty |
| WBC | 3 | 231 | Mean Difference (IV, 95% CI) I^2^=0 | -0.60[-0.88, -0.31] | Very low certainty |
| NEUT% | 4 | 273 | Mean Difference (IV, 95% CI) I^2^=80 | -3.12[-7.63, 1.39] | Very low certainty |
| LYM% | 4 | 213 | Mean Difference (IV, 95% CI) I^2^=23% | 2.69[1.01, 4.36] | Very low certainty |
| **Other outcomes** | | | | | |
| Negative rate of influenza virus nucleic acid | 2 | 178 | Risk Ratio  (M-H, 95% CI) I^2^=0 | 1.27[1.06, 1.52] | Very low certainty |
| CAT Scores | 3 | 227 | Mean Difference (IV, 95% CI) I^2^=8% | -3.08[-3.74, -2.41] | Very low certainty |

**Supplementary Table 8. *Phragmites communis* versus *Control***

| **Outcome or subgroup title** | **No. of studies** | **No. of participants** | **Statistical method**  **(random model)** | **Effect size** | **Quality of**  **Evidence(GRADE)** |
| --- | --- | --- | --- | --- | --- |
| **Symptoms** | | | | |  |
| Cure rate | 15 | 1680 | Risk Ratio (M-H, 95% CI) *I^2^*=45% | 1.57[1.36, 1.81] | Very low certainty |
| Symptomatic drugs | 5 | 350 | Risk Ratio (M-H, 95% CI) *I^2^*=53% | 2.14[1.21, 3.76] | Low certainty |
| Antimicrobial drugs | 10 | 1330 | Risk Ratio (M-H, 95% CI) *I^2^*=45% | 1.53[1.32, 1.77] | Low certainty |
| TCM Syndrome scores | 4 | 239 | Mean Difference  (IV, 95% CI) *I^2^*=72% | -1.21[-2.34, -0.07] | Very low certainty |
| Cure time | 3 | 500 | Std.Mean Difference (IV, 95% CI) *I^2^*=96% | -0.47[-1.56, 0.63] | Very low certainty |
| Antipyretic time | 4 | 773 | Std.Mean Difference (IV, 95% CI) *I^2^*=97% | -1.24[-2.37,-0.11] | Very low certainty |

**Supplementary Table 9** List of abbreviations

| **Abbreviation** | **Meaning** |
| --- | --- |
| ARTIs | Acute Respiratory Tract Infections |
| P. communis | Phragmites communis |
| RCTs | Randomized Controlled Trials |
| RR | Risk Ratios |
| MD | Mean Differences |
| CI | Confidence Intervals |
| AECOPD | Acute Exacerbation of Chronic Obstructive Pulmonary Disease |
| SMD | Standardized Mean Differences |
| TCM | Traditional Chinese Medicine |
| CMCRG | Chinese Medicine Clinical Research Guidelines |
| ICH | International Council on Harmonisation of Technical Requirements for Registration of Pharmaceuticals for Human Use (ICH) |
| CONSORT | Consolidated Standards of Reporting Trials |
| FEV1 | [Forced Expiratory Volume in one second](https://www.healthline.com/health/fev1-copd) |
| FEV1% | Forced Expiratory Volume in one second as a percentage of forced vital capacity |
| FVC | Forced Vital Capacity |
| TNF-α | [Tumor Necrosis Factor-alpha](https://www.biomart.cn/news/16/129807.htm) |
| TGF-β | [Transforming Growth Factor beta](https://en.wikipedia.org/wiki/Transforming_growth_factor_beta) |
| GRO-α | Growth-Regulated Oncogene alpha |
| IL-4 | [Interleukin 4](https://www.genecards.org/cgi-bin/carddisp.pl?gene=IL4) |
| IL-6 | [Interleukin 6](https://www.genecards.org/cgi-bin/carddisp.pl?gene=IL4) |
| IL-8 | [Interleukin 8](https://www.genecards.org/cgi-bin/carddisp.pl?gene=IL4) |
| PCT | [Procalcitonin](https://www.youlai.cn/ask/65A9B7gmVO5.html) |
| WBC | [white blood cell](https://en.wikipedia.org/wiki/White_blood_cell) |
| IL-10 | [Interleukin 1](https://www.genecards.org/cgi-bin/carddisp.pl?gene=IL4)0 |
| LYM% | lymphocyte percentage |
| NEUT | [Neutrophil](https://www.verywellhealth.com/what-are-neutrophils-p2-2249134) |
| SAA | Serum Amyloid A |
| CAT | COPD Assessment Test |
| IL-1 | Interleukin 1 |
| CRP | [C-reactive protein](https://en.wikipedia.org/wiki/C-reactive_protein) |

**Supplementary Table 10 ConPhyMP checklist of Tradition Chinese Medicine of included studies**

| **ID** | **type of extract** | **ConPhyMP checklists table 1** | | | | | **ConPhyMP checklists table 2a/2b/2c** | | | | |
| --- | --- | --- | --- | --- | --- | --- | --- | --- | --- | --- | --- |
|  |  | **Item 1：title and abstract** | **Item 2: Description of the botanical drug and taxonomic authentication** | **Item 3: Description of the extract and extraction process** | **Item 4:Documentation of the legal basis for collection and processing** | **Item 5: Description of product characteristics,in case of a finished (commercial) product** | **Item 1: Type of extract** | **Item 2:Preferred/main methods for**  **extract characterisation/**  **chemical analysis** | **Item 3: Alternative methods for extract**  **characterisation/chemical analysis** | **Item 4: Use of reference standards** | **Item 5: Comparison of different extracts/**  **samples of the same plants** |
| Huai W 2009 | A | *Yes* | *No* | *No* | *No* | *No* | *Yes* | *No* | *No* | *No* | *No* |
| Wang QM 2010 | A | *Yes* | *No* | *No* | *No* | *No* | *Yes* | *No* | *No* | *No* | *No* |
| Zhang XT 2016 | A | *Yes* | *No* | *No* | *No* | *No* | *Yes* | *No* | *No* | *No* | *No* |
| Zhang MX 2006 | C | *Yes* | *No* | *No* | *No* | *No* | *Yes* | *Not Applicable* | *No* | *No* | *No* |
| Xu XF 2013 | C | *Yes* | *No* | *No* | *No* | *No* | *Yes* | *Not Applicable* | *No* | *No* | *No* |
| Sun JF-1 2014 | C | *Yes* | *No* | *No* | *No* | *No* | *Yes* | *Not Applicable* | *No* | *No* | *No* |
| Sun JF-2 2014 | B | *Yes* | *No* | *No* | *No* | *No* | *Yes* | *No* | *No* | *No* | *No* |
| Song DS 2021 | C | *Yes* | *No* | *No* | *No* | *No* | *Yes* | *Not Applicable* | *No* | *No* | *No* |
| Xue XF 2012 | A | *Yes* | *No* | *No* | *No* | *No* | *Yes* | *No* | *No* | *No* | *No* |
| Sun L 2022 | C | *Yes* | *No* | *No* | *No* | *No* | *Yes* | *Not Applicable* | *No* | *No* | *No* |
| Li YP 2022 | C | *Yes* | *No* | *No* | *No* | *No* | *Yes* | *Not Applicable* | *No* | *No* | *No* |
| Zhou ZW 2021 | C | *Yes* | *No* | *No* | *No* | *No* | *Yes* | *Not Applicable* | *No* | *No* | *No* |
| Li J 2020 | C | *Yes* | *No* | *No* | *No* | *No* | *Yes* | *Not Applicable* | *No* | *No* | *No* |
| Luo HL 2019 | C | *Yes* | *No* | *No* | *No* | *No* | *Yes* | *Not Applicable* | *No* | *No* | *No* |
| Hu QY 2019 | C | *Yes* | *No* | *No* | *No* | *No* | *Yes* | *Not Applicable* | *No* | *No* | *No* |
| Yang LY 2019 | C | *Yes* | *No* | *No* | *No* | *No* | *Yes* | *Not Applicable* | *No* | *No* | *No* |
| Xue JH 2017 | C | *Yes* | *No* | *No* | *No* | *No* | *Yes* | *Not Applicable* | *No* | *No* | *No* |
| Zhang ZX 2017 | C | *Yes* | *No* | *No* | *No* | *No* | *Yes* | *Not Applicable* | *No* | *No* | *No* |
| Cheng Y 2016 | C | *Yes* | *No* | *No* | *No* | *No* | *Yes* | *Not Applicable* | *No* | *No* | *No* |
| Sun YT 2014 | C | *Yes* | *No* | *No* | *No* | *No* | *Yes* | *Not Applicable* | *No* | *No* | *No* |
| Wei ZJ 2014 | C | *Yes* | *No* | *No* | *No* | *No* | *Yes* | *Not Applicable* | *No* | *No* | *No* |
| Pan XS 2014 | B | *Yes* | *No* | *No* | *No* | *Yes* | *Yes* | *No* | *No* | *No* | *No* |
| Zhou JC 2013 | B | *Yes* | *No* | *No* | *No* | *Yes* | *Yes* | *No* | *No* | *No* | *No* |
| Wang FW 2007 | C | *Yes* | *No* | *No* | *No* | *No* | *Yes* | *Not Applicable* | *No* | *No* | *No* |
| Li CY 2022 | C | *Yes* | *No* | *No* | *No* | *No* | *Yes* | *Not Applicable* | *No* | *No* | *No* |
| Li HY 2022 | C | *Yes* | *No* | *No* | *No* | *No* | *Yes* | *Not Applicable* | *No* | *No* | *No* |
| Zheng YL 2019 | C | *Yes* | *No* | *No* | *No* | *No* | *Yes* | *Not Applicable* | *No* | *No* | *No* |
| Zhang ZX 2016 | C | *Yes* | *No* | *No* | *No* | *No* | *Yes* | *Not Applicable* | *No* | *No* | *No* |
| Chen H 2014 | C | *Yes* | *No* | *No* | *No* | *No* | *Yes* | *Not Applicable* | *No* | *No* | *No* |
| Hui P 2012 | C | *Yes* | *No* | *No* | *No* | *No* | *Yes* | *Not Applicable* | *No* | *No* | *No* |
| Shi JP 2010 | C | *Yes* | *No* | *No* | *No* | *No* | *Yes* | *Not Applicable* | *No* | *No* | *No* |
| Wong 2012 | A | *Yes* | *No* | *No* | *No* | *No* | *Yes* | *No* | *No* | *No* | *No* |
| Hong LL 2020 | C | *Yes* | *No* | *No* | *No* | *No* | *Yes* | *Not Applicable* | *No* | *No* | *No* |
| Wang DY 2020 | C | *Yes* | *No* | *No* | *No* | *No* | *Yes* | *Not Applicable* | *No* | *No* | *No* |
| Ye D 2010 | C | *Yes* | *No* | *No* | *No* | *No* | *Yes* | *Not Applicable* | *No* | *No* | *No* |
| Bi DX 2019 | C | *Yes* | *No* | *No* | *No* | *No* | *Yes* | *Not Applicable* | *No* | *No* | *No* |
| Guo XH 2009 | A | *Yes* | *No* | *No* | *No* | *No* | *Yes* | *No* | *No* | *No* | *No* |
| Zong Y 2020 | C | *Yes* | *No* | *No* | *No* | *No* | *Yes* | *Not Applicable* | *No* | *No* | *No* |
| Chen XM 2016 | C | *Yes* | *No* | *No* | *No* | *No* | *Yes* | *Not Applicable* | *No* | *No* | *No* |
| Guo XX 2017 | C | *Yes* | *No* | *No* | *No* | *No* | *Yes* | *Not Applicable* | *No* | *No* | *No* |
| Chang QJ 2016 | C | *Yes* | *No* | *No* | *No* | *No* | *Yes* | *Not Applicable* | *No* | *No* | *No* |
| Xu GJ 2014 | C | *Yes* | *No* | *No* | *No* | *No* | *Yes* | *Not Applicable* | *No* | *No* | *No* |
| Li HJ 2022 | A | *Yes* | *No* | *No* | *No* | *No* | *Yes* | *No* | *No* | *No* | *No* |

## Supplementary Figures


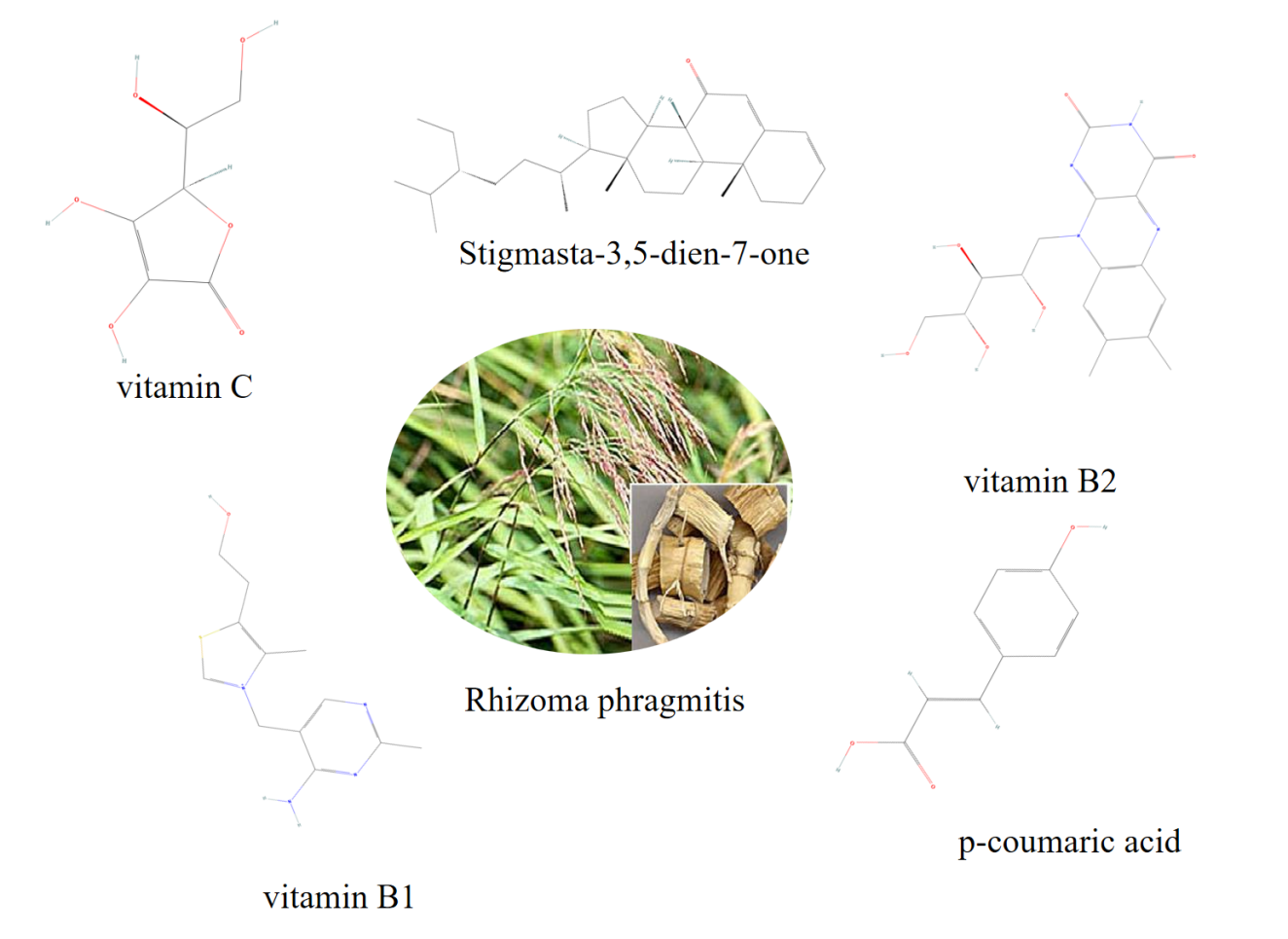


**Supplementary Figure 1** The secondary metabolites isolated from Phragmites communis

**
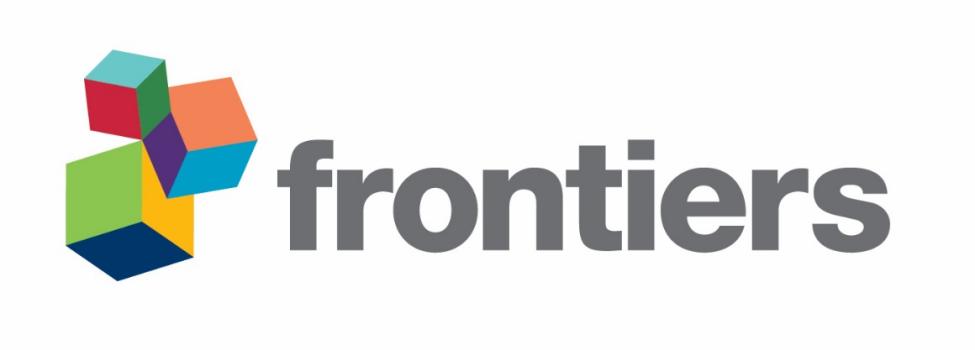
**
